# Outcomes of SARS-CoV-2 Omicron Variant Infections Compared With Seasonal Influenza and Respiratory Syncytial Virus Infections in Adults Attending the Emergency Department: A Multicenter Cohort Study

**DOI:** 10.1093/cid/ciad660

**Published:** 2023-10-26

**Authors:** Pontus Hedberg, John Karlsson Valik, Lina Abdel-Halim, Tobias Alfvén, Pontus Nauclér

**Affiliations:** Department of Medicine Huddinge, Karolinska Institutet, Stockholm, Sweden; Division of Infectious Diseases, Department of Medicine, Solna, Karolinska Institutet, Stockholm, Sweden; Department of Infectious Diseases, Karolinska University Hospital, Stockholm, Sweden; Division of Infectious Diseases, Department of Medicine, Solna, Karolinska Institutet, Stockholm, Sweden; Department of Infectious Diseases, Karolinska University Hospital, Stockholm, Sweden; Department of Global Public Health, Karolinska Institutet, Stockholm, Sweden; Sachs’ Children and Youth Hospital, Stockholm, Sweden; Division of Infectious Diseases, Department of Medicine, Solna, Karolinska Institutet, Stockholm, Sweden; Department of Infectious Diseases, Karolinska University Hospital, Stockholm, Sweden

**Keywords:** SARS-CoV-2, Omicron, influenza, respiratory syncytial virus, emergency department

## Abstract

**Background:**

There is a controversy over the impact of severe acute respiratory syndrome coronavirus 2 (SARS-CoV-2) infections in an era of less virulent variants and an increasing population immunity. We compared outcomes in adults attending the emergency department (ED) with an Omicron, influenza, or respiratory syncytial virus (RSV) infection.

**Methods:**

Retrospective multicenter cohort study including adults attending the ED in 6 acute care hospitals in Stockholm County, Sweden, with an Omicron, influenza, or RSV infection during 2021–2022 and 2015–2019. During 2021–2022, patients were tested for all 3 viruses by multiplex polymerase chain reaction (PCR) testing. The primary outcome was 30-day all-cause mortality. Secondary outcomes were 90-day all-cause mortality, hospitalization, and intensive care unit (ICU) admission.

**Results:**

A total of 6385 patients from 2021–2022 were included in the main analyses: 4833 Omicron, 1099 influenza, and 453 RSV. The 30-day mortality was 7.9% (n = 381) in the Omicron, 2.5% (n = 28) in the influenza, and 6.0% (n = 27) in the RSV cohort. Patients with Omicron had an adjusted 30-day mortality odds ratio (OR) of 2.36 (95% confidence interval [CI] 1.60–3.62) compared with influenza and 1.42 (95% CI .94–2.21) compared with RSV. Among unvaccinated Omicron patients, stronger associations were observed compared with both influenza (OR 5.51 [95% CI 3.41–9.18]) and RSV (OR 3.29 [95% CI 2.01–5.56]). Similar trends were observed for secondary outcomes. Findings were consistent in comparisons with 5709 pre-pandemic influenza 995 RSV patients.

**Conclusions:**

In patients attending the ED, infections with Omicron were both more common and associated with more severe outcomes compared with influenza and RSV, in particular among unvaccinated patients.

## Teaser

Adults attending the emergency department with an Omicron infection had more severe outcomes compared with patients with influenza, particularly among unvaccinated individuals. For respiratory syncytial virus, differences were less evident, but clear when restricting the Omicron cohort to unvaccinated individuals.

Compared with previous severe acute respiratory syndrome coronavirus 2 (SARS-CoV-2) variants, Omicron has had wider societal dissemination but a milder clinical course with reduced mortality rates [1, 2]. The evolving nature of SARS-CoV-2, with later strains exhibiting less severe manifestations, suggests a potential convergence with respiratory syncytial virus (RSV) and influenza in terms of clinical presentation. During earlier stages of the SARS-CoV-2 pandemic, the incidence of RSV and influenza declined [3]. Both RSV and seasonal influenza are long-standing public health concerns, causing substantial morbidity and mortality among the elderly and those with certain chronic medical conditions [4, 5]. During 2021 and 2022, the incidence of RSV and influenza resurged, likely a consequence of changes in coronavirus disease 2019 (COVID-19) control strategies [6, 7].

Studies comparing SARS-CoV-2 with influenza have demonstrated higher mortality rates associated with SARS-CoV-2 infections [8–10]. However, many of these studies focused on earlier variants of SARS-CoV-2 when SARS-CoV-2 vaccination strategies were yet to be fully implemented. Comparisons between the Omicron variant, influenza, and in particular RSV, remain limited in the current body of research, leaving important questions unanswered. This includes understanding outcomes of Omicron in relation to RSV, an under-recognized cause of deterioration in elderly patients, with recommendations of RSV vaccination in older adults being issued in both Europe and the United States [11–13].

Previous studies comparing these viruses have often been hampered by differential testing strategies, thereby risking bias [14]. We investigated outcomes for Omicron compared with seasonal influenza and RSV during the 2021/2022 season in a cohort of patients attending hospital emergency departments (ED), where patients were extensively tested for all 3 viruses, as well as pre-pandemic seasons (2015–2019) of influenza and RSV.

## METHODS

### Study Design and Data Sources

We conducted a retrospective multicentre cohort study in adults attending an ED at 6 hospitals in Stockholm County, Sweden, with an Omicron, seasonal influenza, or RSV infection. Data were linked using personal identification numbers from the TakeCare® Intelligence database, the Stockholm regional healthcare data warehouse, Statistics Sweden, SmiNet, the National Vaccination Register, the Swedish Intensive Care Registry, and the SARS-CoV-2 national quality registry. These data sources contained all microbiological tests performed in patients attending 6 acute care hospitals, sociodemographic data, drug prescriptions, inpatient stays, outpatient visits, all SARS-CoV-2 positive polymerase chain reaction (PCR) tests, serology testing, COVID-19 vaccinations, intensive care unit (ICU) admissions, and deaths (Supplementary Data).

### Study Population

#### 2021 to 2022 Cohorts

We identified all ED visits from individuals ≥18 years in 6 acute care hospitals from 1 August 2021 to 15 September 2022. These dates included patients from the start of the influenza and RSV seasons 2021/2022 and enabled a 30-day follow-up (data available until 15 October 2022). We only considered visits from patients who had lived in Stockholm County ≥1 year before the visit to enable classification of baseline characteristics. Among these visits, we identified all visits with a PCR test positive for SARS-CoV-2, influenza A/B, or RSV any time from 14 days before to 1 day after the visit. Patients positive for multiple viruses were excluded. Multiplex PCR testing of SARS-CoV-2, influenza A/B, and RSV was introduced February 2021, meaning almost all patients in the ED tested for 1 of the viruses were also tested for the other viruses simultaneously. We restricted the Omicron cohort to patients attending the ED from 27 December 2021 and onward, a time period when Omicron was the dominant variant of concern in Sweden [15]. Furthermore, to restrict the study population to individuals attending the ED due to the respiratory virus infection, 2 senior infectious disease physicians reviewed all ED codes to only include visits likely to be caused by the virus infection (Supplementary Table 1). For the final study cohort, we included the first such visit from each patient.

#### Pre-pandemic Cohorts (2015 to 2019)

To also compare characteristics and outcomes of the Omicron cohort with pre-pandemic influenza and RSV cohorts, we identified all patients attending the ED with influenza or RSV from 1 August 2015 to 31 December 2019. These analyses were performed to account for potential differences in diseases severity of influenza and RSV between seasons. The same inclusion procedure was used as described above.

### Outcomes and Other Variables

The primary outcome was 30-day all-cause mortality. Secondary outcomes were 90-day all-cause mortality, hospitalization, and ICU admission. Hospitalizations with a main ICD-10 diagnosis code indicative of admission due to the respiratory virus infection were considered. These codes were reviewed by 2 senior infectious disease physicians (Supplementary Table 2). Patients were followed for hospitalization up to 14 days after the visit.

Data on biological sex, age, region of birth, education level, comorbidities, number of COVID-19 vaccine doses received up until 14 days before the visit, and positive SARS-CoV-2 PCR tests more than 90 days before the visit or serology tests until date of first vaccination (indicating a previous infection) were collected. Comorbidities were based on conditions with an increased risk of severe COVID-19 [16]. The variables are further described in Supplementary Table 3.

### Statistical Methods

We described characteristics of the patients in the cohorts. The crude cumulative incidence of 30-day and 90-day all-cause mortality was analyzed using the Aalen and Johansen estimator [17]. We then described crude rates of hospitalization, ICU admission, 30-day and 90-day all-cause mortality in the overall cohorts, as well as stratifying the Omicron cohort into unvaccinated (zero doses) and vaccinated (2 doses or more) patients. Crude and adjusted regression models were fitted to compare the outcomes of the Omicron cohort with the other cohorts. Logistic regression models were used for 30-day mortality and 90-day mortality, whereas Cox proportional hazards models were used for hospitalization and ICU admission. The models were adjusted for age (included as a continuous variable using restricted cubic splines with 4 knots [18]), sex, being born in Sweden or not, education level, and all studied comorbidities. Region of birth and education level were missing for 0.02% and 4.6% of the study population, respectively, and these individuals were included in all analyses classified as missing. Complete information was available for all other variables. For the primary outcome, 30-day all-cause mortality, the adjusted model was also used to compare the odds among unvaccinated and previously SARS-CoV-2 uninfected with the influenza 2021/2022 and the RSV 2021/2022 cohort, respectively. Furthermore, to better understand the effect of vaccination in the Omicron cohort, models adjusted for age, sex, being born in Sweden or not, education level, and all studied comorbidities were used to compare the odds of 30-day mortality in those with 1, 2, 3, or 4 doses, respectively, compared with unvaccinated patients. To investigate a potential effect modification by age, likelihood ratio tests were used to compare models including age and virus exposure with or without an interaction term. Age-stratified crude outcome rates were also described for the cohorts. For 90-day all-cause mortality, the analyses were restricted to visits up until 17 July 2022 to enable a 90-day follow-up. A sensitivity analysis with all ED visits were performed to better understand if the exclusion of visits without a code indicative of virus infection would lead to different findings compared with including all visits. The first such visit were included from each patient. Characteristics of visits with and without such a diagnosis were compared. All analyses were conducted using R version 4.1.0.

## RESULTS

### Study Population

**Figure 1.**
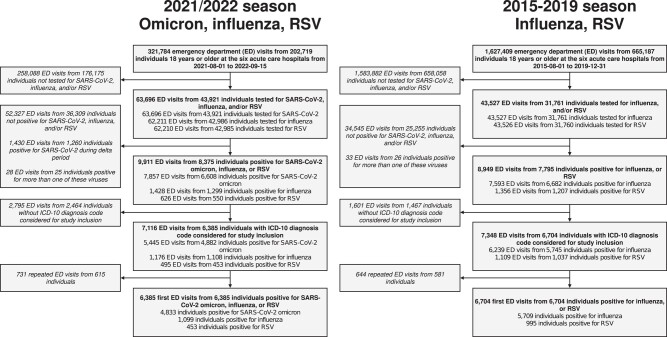
Study flow chart for the study population during the 2021/2022 season and 2015–2019 seasons. Abbreviations: ED, emergency department; ICD-10, International Statistical Classification of Diseases and Related Health Problems 10th Revision; RSV, respiratory syncytial virus; SARS-CoV-2, severe acute respiratory syndrome coronavirus 2.

**Figure 2.**
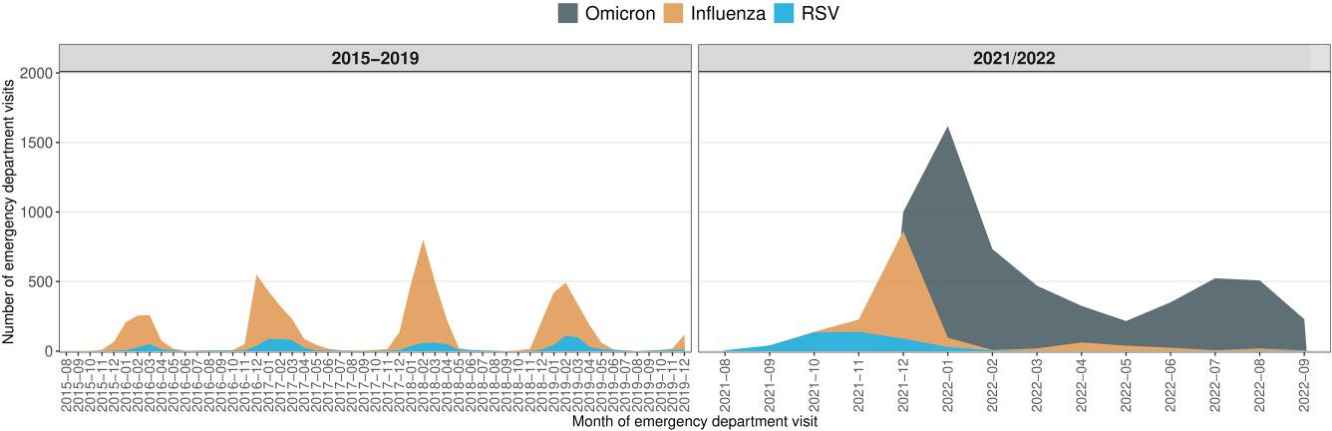
Stacked area chart of number of patients included per calendar month. Abbreviation: RSV, respiratory syncytial virus.

Of 63,696 2021/2022 ED visits tested for SARS-CoV-2, influenza, or RSV, 62 210 (98%) were tested for all 3 viruses (Figure 1). Of the 9911 visits from 8375 individuals positive for any of the viruses, 6385 visits with a diagnostic code indicative of respiratory infection from the same number of patients were included in the study analyses for the 2021/2022 season: 4833 Omicron, 1099 influenza (1082 influenza A), and 453 RSV. A total of 5709 pre-pandemic influenza patients (4214 influenza A) and 995 pre-pandemic RSV patients were included in the study analyses. The monthly number of patients included is presented in Figure 2 and the distribution over the Omicron period is presented in Supplementary Figure 1.

### Baseline Characteristics

In the cohorts from 2021/2022, patients with Omicron and RSV were older compared with influenza, with 42.0% (n = 2030) being 75 years or older in the Omicron cohort, compared with 28.4% (n = 312) in the influenza cohort, and 45.5% (n = 206) in the RSV cohort (Table 1). In the pre-historic influenza and RSV cohorts 37.7% (n = 2150) and 53.4% (n = 531) were 75 years or older. Hypertension, followed by cardiac or cerebrovascular disease, were the most common comorbidities in all cohorts. For most comorbidities, the prevalence was highest among patients with RSV. A total of 22.1% (n = 1068) of the Omicron cohort were unvaccinated before the visit, and 75% (n = 3625) had received 2 doses or more. Characteristics of the Omicron cohort stratified on number of vaccine doses before the visit is presented in Supplementary Table 4. Overall, the age and number of comorbidities increased with the number of vaccine doses. Patients with a diagnosis indicative of virus infection tended to more often have chronic lung disease and being immunocompromised compared with patients without such a diagnosis (Supplementary Tables 5–9).

### 30-day and 90-day All-cause Mortality

**Figure 3.**
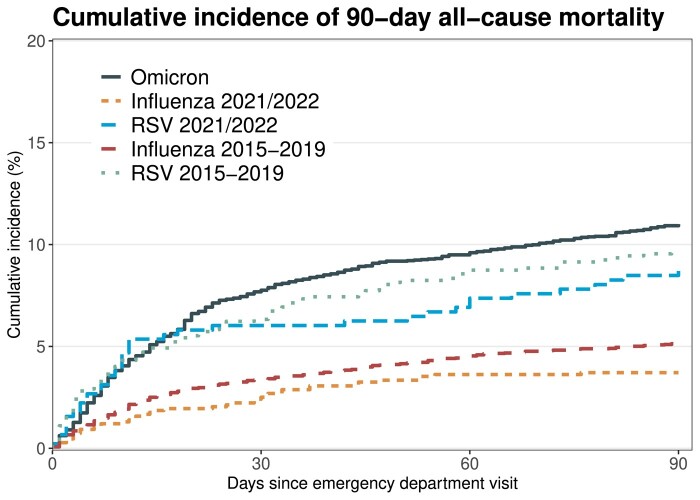
Cumulative incidence plot for 90-day all-cause mortality in the cohorts. This analysis was restricted to patients attending the emergency department up until 17 July 2022 to enable for 90 d of follow-up. Abbreviation: RSV, respiratory syncytial virus.

The 30-day mortality was 7.9% (n = 381) in the Omicron cohort, 2.5% (n = 28) in the influenza 2021/2022 cohort, 6.0% (n = 27) in the RSV 2021/2022 cohort, 3.4% (n = 192) in the influenza pre-pandemic cohort, and 6.3% (n = 63) in the RSV pre-pandemic cohort (Figure 3 and Table 2). The mortality was similar in the sensitivity analysis including visits both with and without a diagnosis code indicating a respiratory virus infection (Supplementary Figure 1). During the 2021/2022 season, the 30-day mortality was 2.6% (n = 28) for patients infected with influenza A, whereas none of the 17 patients infected with influenza B died. During the 2015–2019 seasons, the 30-day mortality rate was 3.2% (n = 135) in patients with influenza A and 3.8% (n = 57) in patients with influenza B.

**Table 1.** Characteristics of the Cohorts

Variable	Omicron (n = 4833)	Influenza 2021/2022 (n = 1099)	RSV 2021/2022 (n = 453)	Influenza 2015–2019 (n = 5709)	RSV 2015–2019 (n = 995)
Male sex	2459 (50.9)	474 (43.1)	207 (45.7)	2628 (46.0)	420 (42.2)
Age, y	70.0 [51.0, 81.0]	56.0 [32.0, 77.0]	72.0 [57.0, 82.0]	68.0 [47.0, 80.0]	76.0 [63.0, 85.0]
18–44	960 (19.9)	453 (41.2)	72 (15.9)	1300 (22.8)	92 (9.2)
45–54	440 (9.1)	82 (7.5)	33 (7.3)	582 (10.2)	49 (4.9)
55–64	636 (13.2)	105 (9.6)	54 (11.9)	693 (12.1)	129 (13.0)
65–74	767 (15.9)	147 (13.4)	88 (19.4)	984 (17.2)	194 (19.5)
75 or older	2030 (42.0)	312 (28.4)	206 (45.5)	2150 (37.7)	531 (53.4)
Region of birth					
Africa	176 (3.6)	67 (6.1)	15 (3.3)	240 (4.2)	30 (3.0)
The Americas	139 (2.9)	41 (3.7)	8 (1.8)	152 (2.7)	21 (2.1)
Asia or Oceania	705 (14.6)	249 (22.7)	66 (14.6)	866 (15.2)	87 (8.7)
Europe	651 (13.5)	122 (11.1)	79 (17.4)	798 (14.0)	132 (13.3)
Sweden	3162 (65.4)	620 (56.4)	285 (62.9)	3650 (63.9)	725 (72.9)
Missing	0 (0.0)	0 (0.0)	0 (0.0)	3 (0.1)	0 (0.0)
Education level					
Primary	1248 (25.8)	328 (29.8)	119 (26.3)	1490 (26.1)	294 (29.5)
Secondary	1876 (38.8)	418 (38.0)	161 (35.5)	2299 (40.3)	386 (38.8)
Tertiary	1444 (29.9)	293 (26.7)	147 (32.5)	1710 (30.0)	271 (27.2)
Missing	265 (5.5)	60 (5.5)	26 (5.7)	210 (3.7)	44 (4.4)
Cancer	553 (11.4)	84 (7.6)	61 (13.5)	537 (9.4)	149 (15.0)
Cardiac or cerebrovascular disease	1722 (35.6)	286 (26.0)	177 (39.1)	1805 (31.6)	467 (46.9)
Chronic kidney failure	679 (14.0)	101 (9.2)	73 (16.1)	450 (7.9)	117 (11.8)
Chronic liver disease	133 (2.8)	26 (2.4)	17 (3.8)	137 (2.4)	26 (2.6)
Chronic lung disease	697 (14.4)	149 (13.6)	116 (25.6)	893 (15.6)	274 (27.5)
Diabetes	1009 (20.9)	167 (15.2)	111 (24.5)	976 (17.1)	215 (21.6)
Home care services or nursing home	1327 (27.5)	188 (17.1)	125 (27.6)	1094 (19.2)	302 (30.4)
Hypertension	2336 (48.3)	394 (35.9)	242 (53.4)	2349 (41.1)	529 (53.2)
Immunocompromised state	902 (18.7)	125 (11.4)	92 (20.3)	882 (15.4)	232 (23.3)
Neurologic conditions, including dementia	548 (11.3)	69 (6.3)	40 (8.8)	534 (9.4)	118 (11.9)
Obesity	461 (9.5)	118 (10.7)	56 (12.4)	385 (6.7)	67 (6.7)
COVID-19 vaccine doses					
Unvaccinated	1068 (22.1)	214 (19.5)	46 (10.2)	NA	NA
1 dose	140 (2.9)	51 (4.6)	20 (4.4)	NA	NA
2 doses	1128 (23.3)	507 (46.1)	278 (61.4)	NA	NA
3 doses	1740 (36.0)	295 (26.8)	106 (23.4)	NA	NA
4 doses	757 (15.7)	32 (2.9)	3 (0.7)	NA	NA
Previous SARS-CoV-2 infection	389 (8.0)	242 (22.0)	77 (17.0)	NA	NA

Abbreviations: COVID-19, coronavirus disease 2019; NA, not applicable; RSV, respiratory syncytial virus; SARS-CoV-2, severe acute respiratory syndrome coronavirus 2.

**Table 2.** Outcomes for the Omicron Cohort Compared With the Influenza and RSV Cohorts

	Descriptive Data			Omicron Versus Influenza		Omicron Versus RSV	
	Omicron	Influenza	RSV	Unadjusted Ratio (95% CI)	Adjusted Ratio^a^ (95% CI)	Unadjusted Ratio (95% CI)	Adjusted Ratio^a^ (95% CI)
30-day all-cause mortality^b^							
Main cohorts	381/4833 (7.9)	28/1099 (2.5)	27/453 (6.0)	3.27 (2.26–4.94)	2.36 (1.60–3.62)	1.35 (.92–2.07)	1.42 (.94–2.21)
Unvaccinated Omicron^c^	90/1068 (8.4)	28/1099 (2.5)	27/453 (6.0)	3.52 (2.31–5.52)	5.51 (3.41–9.18)	1.45 (.94–2.30)	3.29 (2.01–5.56)
Vaccinated Omicron^d^	283/3625 (7.8)	28/1099 (2.5)	27/453 (6.0)	3.24 (2.22–4.91)	2.00 (1.35–3.10)	1.34 (.91–2.05)	1.20 (.79–1.88)
Pre-pandemic influenza and RSV	381/4833 (7.9)	192/5709 (3.4)	63/995 (6.3)	2.46 (2.06–2.94)	2.17 (1.80–2.62)	1.27 (.97–1.68)	1.49 (1.12–2.01)
90-day all-cause mortality^b,e^							
Main cohorts	424/3854 (11.0)	40/1077 (3.7)	39/453 (8.7)	3.15 (2.31–4.43)	2.31 (1.65–3.30)	1.30 (.93–1.85)	1.40 (.98–2.03)
Unvaccinated Omicron^c^	94/942 (10.0)	40/1077 (3.7)	39/453 (8.7)	2.87 (1.98–4.25)	4.94 (3.17–7.88)	1.18 (.81–1.75)	2.72 (1.75–4.31)
Vaccinated Omicron^d^	318/2797 (11.4)	40/1077 (3.7)	39/453 (8.7)	3.33 (2.40–4.72)	2.17 (1.53–3.16)	1.35 (.96–1.93)	1.27 (.88–1.88)
Pre-pandemic influenza and RSV	424/3854 (11.0)	293/5709 (5.1)	96/995 (9.6)	2.28 (1.96–2.67)	2.09 (1.77–2.48)	1.16 (.92–1.47)	1.43 (1.12–1.85)
Hospital admission^f^							
Main cohorts	2449/4833 (50.7)	435/1099 (39.6)	291/453 (64.2)	1.37 (1.24–1.52)	0.97 (.88–1.08)	0.68 (.60–.77)	0.73 (.64–.82)
Unvaccinated Omicron^c^	494/1068 (46.3)	435/1099 (39.6)	291/453 (64.2)	1.21 (1.07–1.38)	1.30 (1.14–1.49)	0.60 (.52–.70)	0.92 (.79–1.08)
Vaccinated Omicron^d^	1900/3625 (52.4)	435/1099 (39.6)	291/453 (64.2)	1.44 (1.30–1.60)	0.90 (.81–1.00)	0.71 (.63–.81)	0.68 (.60–.78)
Pre-pandemic influenza and RSV	2449/4833 (50.7)	3024/5709 (53.0)	668/995 (67.1)	0.93 (.88–.98)	0.84 (.79–.89)	0.64 (.59–.70)	0.75 (.69–.82)
ICU admission^f^							
Main cohorts	140/4833 (2.9)	11/1099 (1.0)	10/453 (2.2)	2.93 (1.59–5.42)	2.49 (1.34–4.63)	1.32 (.69–2.50)	1.40 (.74–2.67)
Unvaccinated Omicron^c^	54/1068 (5.1)	11/1099 (1.0)	10/453 (2.2)	5.16 (2.70–9.87)	4.96 (2.56–9.60)	2.31 (1.18–4.54)	2.82 (1.39–5.73)
Vaccinated Omicron^d^	83/3625 (2.3)	11/1099 (1.0)	10/453 (2.2)	2.31 (1.23–4.34)	1.88 (.98–3.61)	1.04 (.54–2.00)	1.08 (.56–2.10)
Pre-pandemic influenza and RSV	140/4833 (2.9)	186/5709 (3.3)	50/995 (5.0)	0.89 (.71–1.10)	0.86 (.69–1.07)	0.57 (.41–.78)	0.58 (.41–.80)

Abbreviations: COVID-19, coronavirus disease 2019; CI, confidence interval; ICU, intensive care unit; RSV, respiratory syncytial virus.

^a^Adjusted for age, sex, being born in Sweden, education level, and all studied comorbidities.

^b^Analysed with logistic regression models.

^c^Defined as having received zero COVID-19 vaccine doses 14 days or more before the emergency department visit.

^d^Defined as having received 2 or more COVID-19 vaccine doses 14 days or more before the emergency department visit.

^e^Restricted to individuals visiting the emergency department up until 17 July 2022 to allow for 90-days of follow-up.

^f^Analysed with Cox proportional hazard regression models.

**Figure 4.**
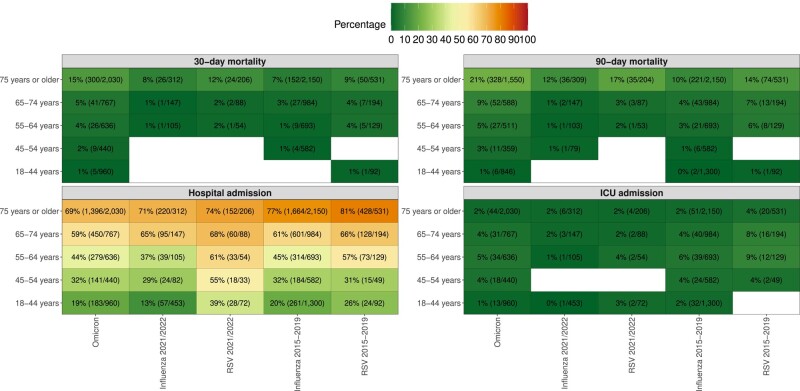
Age-stratified outcome rates in the cohorts. The numbers in each cell represent the percentage experiencing the outcome (number of individuals experiencing the outcome/number of patients in each stratum). The analysis of 90-day all-cause mortality was restricted to patients attending the emergency department up until 17 July 2022 to enable for 90 d of follow-up. Abbreviations: ICU, intensive care unit; RSV, respiratory syncytial virus.

The Omicron cohort had an increased odds of 30-day mortality compared with both the influenza and the RSV cohorts in adjusted models. The adjusted odds ratio (OR) (95% confidence interval [CI]) compared with the influenza and the RSV 2021/2022 cohorts were 2.36 (1.60–3.62) and 1.42 (0.94–2.21), respectively. These odds were for the unvaccinated Omicron cohort 5.51 (3.41–9.18) and 3.29 (2.01–5.56), respectively. These odds were almost identical when restricting the Omicron cohort to unvaccinated and previously SARS-CoV-2 uninfected patients (5.66 [95% CI 3.48–9.49] and 3.38 [95% CI 2.04–5.74], respectively). Among Omicron patients having received 2 doses or more, these odds were 2.00 (1.35–3.10) compared with influenza and 1.20 (0.79–1.88) compared with RSV. When investigating the adjusted OR (95% CI) of 30-day mortality in the Omicron cohort compared with unvaccinated patients, it was 0.66 (0.27–1.40) for 1 dose, 0.42 (0.28–0.62) for 2 doses, 0.42 (0.30–0.58) for 3 doses, and 0.30 (0.21–0.44) for 4 doses. The 30-day mortality among patients ≥75 years was 15% (n = 300) in the Omicron cohort, 8% (n = 26) in the influenza 2021/2022 cohort, 12% (n = 24) in the RSV 2021/2022 cohort, 7% (n = 152) in the influenza pre-pandemic cohort, and 9% (n = 50) in the RSV pre-pandemic cohort (Figure 4). No significant interactions were observed with regards to age and virus exposure when comparing Omicron and influenza (*P* value .14) and Omicron and RSV (*P* value .67). At 90 days, the mortality was 11.0% (n = 424) in the Omicron cohort, 3.7% (n = 40) in the influenza 2021/2022 cohort, 8.7% (n = 39) in the RSV 2021/2022 cohort, 5.1% (n = 293) in the influenza pre-pandemic cohort, and 9.6% (n = 96) in the RSV pre-pandemic cohort (Figure 3 and Table 2). The adjusted odds were similar to those observed for 30-day all-cause mortality. Results from the sensitivity analysis were consistent but showed weaker associations when including all visits, regardless of registered respiratory infection diagnoses (Supplementary Tables 10 and 11).

### Hospitalization and ICU Admission

Hospitalization rates were 50.7% (n = 2449) in the Omicron cohort compared with 39.6% (n = 435) in the influenza 2021/2022 cohort and 64.2% (n = 291) in the RSV 2021/2022 cohort (Table 2). Compared with the 2021/2022 cohorts, admission rates were higher in the pre-pandemic influenza cohort, 53.0% (n = 3024), and RSV cohort, 67.1% (n = 668). No difference in adjusted hazard of hospitalization compared with the influenza 2021/2022 cohort was observed (hazard ratio [HR] 0.97, 95% CI .88–1.08), whereas a reduced adjusted hazard was observed compared with the RSV 2021/2022 cohort (HR 0.73, 95% CI .64–.82). The Omicron cohort had an increased adjusted hazard of ICU admission compared with the influenza 2021/2022 cohort (HR 2.49, 95% CI 1.34–4.63) but not compared with the RSV 2021/2022 cohort (HR 1.40, 95% CI .74–2.67). Overall, besides for ICU admission, a clear increase in each outcome rate was observed with increasing age for all cohorts. Overall, the results were similar, but with overall weaker associations, for the sensitivity analysis including all visits (Supplementary Tables 11 and 12).

## DISCUSSION

Among patients attending the ED, Omicron infections were associated with more severe outcomes compared with influenza infections, particularly among unvaccinated individuals. For RSV infections, such differences were less evident in the overall cohorts, but clear when restricting the Omicron cohort to unvaccinated individuals. Importantly, during the 2021/022 season Omicron infections were both more common and more severe than both influenza and RSV infections. If one would assume that all deaths were related to the respiratory infection and the same length of the infection season, around 14 times more deaths occurred in the Omicron cohort compared with the influenza 2021/2022 cohort and the RSV 2021/2022 cohort, being around 12-fold among patients >75 years and around 30- to 40-fold among patients <75 years.

Studies conducted during earlier stages of the pandemic reported that SARS-CoV-2 exhibited higher disease severity compared with influenza and RSV. We have previously reported 13% 30-day mortality in SARS-CoV-2 infected hospitalized patients compared with 5% and 7% in influenza RSV respectively [9]. A US study showed that 30-day mortality for SARS-CoV-2 was higher than for influenza with an excess death rate of approximately 2% compared with the influenza season of 2022/2023 [19]. However, this study was based on an older and predominantly male veterans cohort, limiting generalizability to other populations. Another Swiss study of hospitalized patients until March 2022 also found increased mortality of SARS-CoV-2 compared with influenza [14]. When restricting their cohort to patients admitted due to SARS CoV-2 or influenza, they too noted a higher proportion of ICU admission in SARS-CoV-2 infected patients. However, further conclusions are hampered by lack of follow-up data after hospital discharge, restricting comparison to a historical influenza cohort, and the inclusion of patients only based on a positive viral test, possibly resulting in many patients primarily being admitted because of other reasons than the viral illness. Both of these studies focused on hospitalized patients, whereas this study considered outcomes in patients attending the ED, irrespectively if they were hospitalized or not. We also had access to complete 90-day follow-up data for all patients, increasing the internal validity of our findings.

With regards to RSV, data comparing adult patient outcomes to the Omicron variant is lacking. A systematic review showed that the rate of mortality and hospitalization did not significantly differ between RSV and influenza, indicating that RSV has a comparable potential to cause severe disease as seasonal influenza [20]. This review is dated from the pre-SARS-CoV-2 era, and it is important to understand the comparative severity in the context of the current pandemic landscape. We show that although the incidence of RSV was lower than both Omicron and influenza, the outcomes were comparable to Omicron, particularly in COVID-19 vaccinated individuals. Development of preventive treatments such as vaccines and monoclonal antibodies against RSV infections have mainly been directed toward infants and children where the burden of disease is high [21]. Recently, 2 RSV vaccine candidates proved to be highly efficient in preventing severe RSV infection in older adults and was approved during late spring in both Europe and the United States [5, 22, 23]. Consequently, in June 2023, agencies in the United States and Europe have issued recommendations of RSV vaccination in older adults, and our findings supports a prompt implementation of the vaccines for the 2023/2024 season [12, 13]. Our findings are not only influenced by the innate virulence of these viruses but also previous immunity acquired from vaccinations and/or previous infections, as well as different clinical management. At least 3 doses of COVID-19 vaccination were recommended to all adults in Sweden, whereas annual influenza vaccination is only recommended to individuals >65 years, those with certain medical risk groups, and certain groups of healthcare professionals.

Strengths of our study include the multicentre design including EDs from six emergency hospitals in Stockholm County, ranging from community hospitals to university hospitals. Furthermore, several population-based data sources with high coverage were used, enabling robust classification of pre-existing comorbidities as well as sociodemographic factors. This is reinforced by the fact that the COVID-19 pandemic has had a differential impact on areas and individuals depending on sociodemographic factors [24]. In addition, the implementation of multiplex PCR testing of all studied viruses in our region in February 2021 reduced the potential selection bias of having differential testing strategies for the three viruses, a major strength compared with other studies.

Limitations include lack of data on influenza vaccinations, precluding vaccination status stratification as was done for the Omicron cohort. The annual influenza vaccine uptake among individuals ≥65 years in Stockholm County ranged from 50% to 52% in 2015% to 2019% and 55% to 62% in 2020 to 2022. Also, we did not extensively distinguish between patients infected with influenza A and B. However, the number of patients infected with influenza B during the 2021/2022 season was low and the 30-day mortality rates were similar for influenza A and B during the 2015–2019 seasons. Furthermore, it is possible that differences in treatment strategies and hospitalization routines of positive patients compared with influenza and RSV positive patients might have had an impact on the observed outcome rates and estimates.

## CONCLUSIONS

In the ED setting where the investigated 3 viruses were tested for simultaneously, Omicron infections were both more common and were associated with more severe outcomes compared with seasonal influenza and RSV infections, particularly among unvaccinated individuals. For RSV, the differences were less evident in the overall cohorts, but clear when restricting the Omicron cohort to unvaccinated individuals. This underscores the need for public health strategies for managing and mitigating the impact of Omicron and other viral respiratory infections, with continued assessments of their comparative severity. Furthermore, it highlights the need of better prevention and treatment options against RSV infection in older adults.

## Supplementary Data

Supplementary materials are available at *Clinical Infectious Diseases* online. Consisting of data provided by the authors to benefit the reader, the posted materials are not copyedited and are the sole responsibility of the authors, so questions or comments should be addressed to the corresponding author.

## Supplementary Material

ciad660_Supplementary_Data

## Notes

***Author contributions.*** P. N. and P. H. had full access to all the data in the study and take responsibility for the integrity of the data and the accuracy of the data analysis. All authors have read and approved the manuscript. Conceptualization: All authors. Data curation: P. H. Formal analysis: P. H. Funding acquisition: P. N. Investigation: All authors. Methodology: All authors. Project administration: All authors. Resources: P. N. Software: P. H. Supervision: P. N. Validation: All authors. Visualization: P. H. Writing original draft: P. H., L. A. H. Writing—review and editing: All authors.

***Data sharing.*** The individual participant data underlying this article were subject to ethical approval and cannot be shared publicly. Data from the deidentified administrative health registry are not freely available due to protection of the personal integrity of the participants.

***Ethics approval and consent to participate.*** The need for consent was waived by the Swedish Ethical Review Authority (Dnr 2018/1030-31, coronavirus disease 2019 (COVID-19) research amendments (Dnr 2020-01385, 2020-02145, 2020-04069, and 2022-02127-02) because analyses are based on retrospectively collected data from the administrative health registry.

***Financial support*.** The work was supported by the Swedish Research Council (Dnr. 2021-04809) and the EuCare Project funded by the European Union’s Horizon Europe Research and Innovation Program under grant agreement number 101046016. The funders had no role in the design and conduct of the study; collection, management, analysis, and interpretation of the data; preparation, review, or approval of the manuscript; and decision to submit the manuscript for publication.
