# Supplementary material for: Outcomes of SARS-CoV-2 Omicron Variant Infections Compared With Seasonal Influenza and Respiratory Syncytial Virus Infections in Adults Attending the Emergency Department: A Multicenter Cohort Study
Source: Clin Infect Dis. 2023 Oct 26;78(4):900–7. doi: 10.1093/cid/ciad660 (PMC11006100; doi:10.1093/cid/ciad660)
Supplement: ciad660_Supplementary_Data [file ciad660_supplementary_data.docx]

Supplementary material for

**Outcomes of SARS-CoV-2 Omicron Variant Infections Compared with Seasonal Influenza and Respiratory Syncytial Virus Infections in Adults Attending the Emergency Department: A Multicentre Cohort Study**

**Authors:** Pontus Hedberg, John Karlsson Valik, Lina Abdel Halim, Tobias Alfvén, Pontus Nauclér

Correspondence to [pontus.hedberg@ki.se](mailto:pontus.hedberg@ki.se)

**Table of contents**

| **Content** | **Page** |
| --- | --- |
| Text S1. Description of data sources | 3-4 |
| Table S1. Emergency department ICD-10 codes used for study inclusion | 5-7 |
| Table S2. Inpatient ICD-10 codes used for classification of hospital admission | 8-11 |
| Table S3. Descriptions of study outcomes and other collected variables | 12-16 |
| Figure S1. Distribution of circulating Omicron sublineages in Stockholm County over the Omicron study period | 17 |
| Table S4. Characteristics of the SARS-CoV-2 omicron study cohort by vaccination status | 18-19 |
| Table S5. Characteristics of individuals with and without an ED diagnosis code indicative of respiratory infection in the Omicron cohort | 20-21 |
| Table S6. Characteristics of individuals with and without an ED diagnosis code indicative of respiratory infection in the influenza 2021/2022 cohort | 22-23 |
| Table S7. Characteristics of individuals with and without an ED diagnosis code indicative of respiratory infection in the RSV 2021/2022 cohort | 24-25 |
| Table S8. Characteristics of individuals with and without an ED diagnosis code indicative of respiratory infection in the influenza 2015-2019 cohort | 26-27 |
| Table S9. Characteristics of individuals with and without an ED diagnosis code indicative of respiratory infection in the RSV 2015-2019 cohort | 28-29 |
| Figure S2. Cumulative incidence plot for 90-day all-cause mortality in the main analysis and the cohorts including all visits | 30 |
| Table S10. Unadjusted regression model ratios in the main analysis compared with a sensitivity analysis including all visits | 31 |
| Table S11. Adjusted regression model ratios in the main analysis compared with a sensitivity analysis including all visits | 32 |
| Supplementary references | 33 |

**Text S1. Descriptions of data sources**

**TakeCare® Intelligence database:** The data from the TakeCare® Intelligence database contained data on all microbiological tests performed in patients attending six acute care hospitals in Stockholm County (Danderyd Hospital, Karolinska University Hospital Huddinge, Karolinska University Hospital Solna, Norrtälje Hospital, Södersjukhuset, and Södertälje Hospital), all using the journal system TakeCare®. The only acute care hospitals in Stockholm County not included in this database is Capio S:t Görans Hospital, using a different journal system. Data on all SARS-CoV-2, influenza A/B, and RSV PCR tests from 1 August 2015 and onwards were used in this study.

**Stockholm regional healthcare data warehouse (VAL):** The Stockholm Regional Council operates the healthcare data warehouse VAL, which contains data from multiple administrative healthcare databases.^1^ This data includes information on inpatient stays, outpatient specialist visits and primary care visits (coverage of around 94%) reimbursed by Region Stockholm.^2^ Furthermore, the data warehouse contains information on demographics, migration status, nursing home residency, home care services, and collected drug prescriptions. Data on all such characteristics for the study population were used in this study.

**Statistics Sweden:** Statistics Sweden is a governmental agency supplying statistics for decision making, debate, and research.^3^ This includes several registers such as the Total Population Register (TPR), and the Integrated Database for Labour Market Research (LISA). Data on region of birth and education level for the study population were used in this study.

**SmiNet:** SmiNet is the electronic system used for surveillance of communicable diseases in Sweden, owned and operated by PHAS and the communicable disease control units in Sweden.^4^ It is used for surveillance of more than 60 notifiable diseases, including COVID-19, according to the Communicable Diseases Act and the Communicable Diseases Ordinance.^5,6^ Data on all polymerase chain reaction (PCR) tests positive for severe acute respiratory syndrome coronavirus 2 (SARS-CoV-2) for the study population were used in this study.

**National Vaccination Register (NVR):** All vaccinations within national vaccination programs and vaccinations against COVID-19 should according to Swedish law be reported to the NVR, which is governed by the Public Health Agency of Sweden (PHAS).^7^ Data on administered COVID-19 vaccine doses for the study population were used in this study.

**Swedish Intensive Care Registry (SIR):** SIR is a national quality register for intensive care which was established in 2001.^8^ SIR prospectively collects data from intensive care unit admission in Sweden, currently including data from all 83 intensive care units in Sweden.^9^ Data on all the intensive care units included in the study population were used in this study to analyze the charactericts and outcomes of the intensive care unit admissions.

**SARS-CoV-2 national quality registry (NKCOV):** The NKCOV registry was set-up to include laboratory test results from all microbiological laboratories that performed analyses for SARS-CoV-2 in Stockholm County. All but one laboratory submitted data to the registry. Data on SARS-CoV-2 serology testing were used in this study.

**Table S1. Emergency department ICD-10 codes used for study inclusion**

| **ICD-10 diagnosis code** | **Diagnosis name** |
| --- | --- |
| A09.0 | Other and unspecified gastroenteritis and colitis of infectious origin |
| A09.9 | Gastroenteritis and colitis of unspecified origin |
| A41.8 | Other specified sepsis |
| A41.9 | Sepsis, unspecified |
| B09.9 | Unspecified viral infection characterized by skin and mucous membrane lesions |
| B34.2 | Coronavirus infection, unspecified site |
| B34.9 | Viral infection, unspecified |
| B97.4 | Respiratory syncytial virus as the cause of diseases classified to other chapters |
| H66.0 | Acute suppurative otitis media |
| H66.9 | Otitis media, unspecified |
| J00.9 | Acute nasopharyngitis |
| J01.0 | Acute maxillary sinusitis |
| J01.1 | Acute frontal sinusitis |
| J01.2 | Acute ethmoidal sinusitis |
| J01.4 | Acute pansinusitis |
| J01.9 | Acute sinusitis, unspecified |
| J02.9 | Acute pharyngitis, unspecified |
| J03.0 | Streptococcal tonsillitis |
| J03.9 | Acute tonsillitis, unspecified |
| J04.0 | Acute laryngitis |
| J04.1 | Acute tracheitis |
| J04.2 | Acute laryngotracheitis |
| J05.0 | Acute obstructive laryngitis |
| J06.8 | Other acute upper respiratory infections of multiple sites |
| J06.9 | Acute upper respiratory infection, unspecified |
| J09.9 | Influenza due to identified zoonotic or pandemic influenza virus |
| J10.0 | Influenza with pneumonia, seasonal influenza virus identified |
| J10.1 | Influenza with other respiratory manifestations, seasonal virus identified |
| J10.8 | Influenza with other manifestations, seasonal influenza virus identified |
| J11.0 | Influenza with pneumonia, virus not identified |
| J11.1 | Influenza with other respiratory manifestations, virus not identified |
| J11.8 | Influenza with other manifestations, virus not identified |
| J12.1 | Respiratory syncytial virus pneumonia |
| J12.2 | Parainfluenza virus pneumonia |
| J12.8 | Other viral pneumonia |
| J12.9 | Viral pneumonia, unspecified |
| J13.9 | Pneumonia due to S. pneumoniae |
| J14.9 | Pneumonia due to Haemophilus influenzae |
| J15.1 | Pneumonia due to Pseudomonas |
| J15.2 | Pneumonia due to staphylococcus |
| J15.4 | Pneumonia due to other streptococci |
| J15.7 | Pneumonia due to Mycoplasma pneumoniae |
| J15.8 | Other bacterial pneumonia |
| J15.9 | Bacterial pneumonia, unspecified |
| J17.0 | Pneumonia in bacterial diseases classified elsewhere |
| J18.0 | Bronchopneumonia, unspecified |
| J18.1 | Lobar pneumonia, unspecified |
| J18.8 | Other pneumonia, organism unspecified |
| J18.9 | Pneumonia, unspecified |
| J20.4 | Acute bronchitis due to parainfluenza virus |
| J20.5 | Acute bronchitis due to respiratory syncytial virus |
| J20.6 | Acute bronchitis due to rhinovirus |
| J20.8 | Acute bronchitis due to other specified organisms |
| J20.9 | Acute bronchitis, unspecified |
| J21.0 | Acute bronchitis due to respiratory syncytial virus |
| J21.9 | Acute bronchitis, unspecified |
| J22.9 | Unspecified acute lower respiratory infection |
| J36.9 | Peritonsillar abscess |
| J39.8 | Other specified diseases of upper respiratory tract |
| J39.9 | Diseases of upper respiratory tract, unspecified |
| J40.9 | Bronchitis not specified as acute or chronic |
| J44.0 | Chronic obstructive pulmonary disease with acute lower respiratory infection |
| J44.1 | Chronic obstructive pulmonary disease with acute exacerbation, unspecified |
| J45.1A | Asthma bronchiale, nonallergic asthma, Acute, infectious |
| J80.9 | Adult respiratory distress syndrome |
| J86.9 | Pyrothorax without fistula |
| J90.9 | Pleural effusion, not elsewhere classified |
| J96.0 | Acute respiratory failure |
| J96.00 | Acute respiratory failure, hypoxia without hypercapnia |
| J96.01 | Acute respiratory failure, hypoxia with hypercapnia |
| J96.9 | Respiratory failure, unspecified |
| J96.90 | Respiratory failure, unspecified, hypoxia without hypercapnia |
| J96.99 | Respiratory failure, unspecified, type unspecified |
| J98.7 | Respiratory infections, not elsewhere classified |
| K52.9W | Noninfective gastroenteritis and colitis, unspecified |
| R04.2 | Haemoptysis |
| R05.9 | Cough |
| R06.0 | Dyspnoea |
| R06.4 | Hyperventilation |
| R06.8 | Other and unspecified abnormalities of breathing |
| R07.1 | Chest pain on breathing |
| R07.3 | Other chest pain |
| R07.4 | Chest pain, unspecified |
| R09.2 | Respiratory arrest |
| R09.8 | Other specified symptoms and signs involving the circulatory and respiratory syndrome |
| R11.9 | Nausea and vomiting |
| R11.9B | Vomiting |
| R50.8 | Other specified fever |
| R50.9 | Fever, unspecified |
| R53.9 | Malaise and fatigue |
| R57.2 | Septic shock |
| R65.1 | Systemic Inflammatory Response Syndrome of infectious origin with organ failure |
| R65.9 | Systemic Inflammatory Response, unspecified |
| R91.9 | Abnormal findings on diagnostic imaging of lung |
| U07.1 | COVID-19, virus identified |
| U07.2 | COVID-19, virus not identified |
| U08.9 | Personal history of COVID-19, unspecified |

**Note:** Both main and secondary diagnosis codes were considered

**Abbreviations:** COVID-19=Coronavirus disease 2019, ICD-10=International Statistical Classification of Diseases and Related Health Problems 10th Revision

**Table S2. Inpatient ICD-10 codes used for classification of hospital admission**

| **ICD-10 diagnosis code** | **Diagnosis name** |
| --- | --- |
| A09.0 | Other and unspecified gastroenteritis and colitis of infectious origin |
| A09.9 | Gastroenteritis and colitis of unspecified origin |
| A40.0 | Sepsis due to streptococcus, group A |
| A40.2 | Sepsis due to streptococcus, group D and eneterococcus |
| A40.3 | Sepsis due to Streptococcus pneumoniae |
| A40.8 | Other streptococcal sepsis |
| A40.9 | Streptococcal sepsis, unspecified |
| A41.0 | Sepsis due to Staphylococcus aureus |
| A41.1 | Sepsis due to other specified staphylococcus |
| A41.2 | Sepsis due to unspecified staphylococcus |
| A41.3 | Sepsis due to Haemophilus influenzae |
| A41.4 | Sepsis due to anaerobes |
| A41.5 | Sepsis due to other Gram-negative organisms |
| A41.8 | Other specified sepsis |
| A41.9 | Sepsis, unspecified |
| A49.0 | Staphylococcal infection, unspecified site |
| A49.1 | Streptococcal and enterococcal infection, unspecified site |
| A49.2 | Haemophilus influenzae infection, unspecified site |
| A49.8 | Other bacterial infections of unspecified site |
| A49.9 | Bacterial infection, unspecified |
| A86.9 | Unspecified viral encephalitis |
| B20.9 | HIV disease resulting in unspecified infectious or parasitic disease |
| B33.8 | Other specified viral disease |
| B34.8 | Other viral infections of unspecified site |
| B34.9 | Viral infection, unspecified |
| B96.5 | Pseudomonas (aeruginosa) as the cause of diseases classified to other chapters |
| B97.2 | Coronavirus as the cause of diseases classified to other chapters |
| B97.4 | Respiratory syncytial virus as the cause of diseases classified to other chapters |
| B99.9 | Other and unspecified infectious diseases |
| G00.1 | Meningitis in bacterial diseases classified elsewhere |
| G03.9 | Meningitis, unspecified |
| G04.8 | Other encephalitis, myelitis and encephalomyelitis |
| G93.3 | Postviral fatigue syndrome |
| G94.3 | Encephalopathy in diseases classified elsewhere |
| H66.0 | Acute suppurative otitis media |
| I30.9 | Acute pericarditis, unspecified |
| I40.8 | Other acute myocarditis |
| I40.9 | Acute myocarditis, unspecified |
| I41.1 | Myocarditis in viral diseases classified elsewhere |
| I46.0 | Cardiac arrest with successful resuscitation |
| I46.9 | Sudden cardiac death, so described |
| I51.4 | Myocarditis, unspecified |
| J00.9 | Acute nasopharyngitis |
| J01.0 | Acute maxillary sinusitis |
| J01.9 | Acute sinusitis, unspecified |
| J03.0 | Streptococcal tonsillitis |
| J03.9 | Acute tonsillitis, unspecified |
| J04.0 | Acute laryngitis |
| J04.1 | Acute tracheitis |
| J04.2 | Acute laryngotracheitis |
| J06.8 | Other acute upper respiratory infections of multiple sites |
| J06.9 | Acute upper respiratory infection, unspecified |
| J09.9 | Influenza due to identified zoonotic or pandemic influenza virus |
| J10.0 | Influenza with pneumonia, seasonal influenza virus identified |
| J10.1 | Influenza with other respiratory manifestations, seasonal virus identified |
| J10.8 | Influenza with other manifestations, seasonal influenza virus identified |
| J11.0 | Influenza with pneumonia, virus not identified |
| J11.1 | Influenza with other respiratory manifestations, virus not identified |
| J11.8 | Influenza with other manifestations, virus not identified |
| J12.1 | Respiratory syncytial virus pneumonia |
| J12.2 | Parainfluenza virus pneumonia |
| J12.8 | Other viral pneumonia |
| J12.9 | Viral pneumonia, unspecified |
| J13.9 | Pneumonia due to S. pneumoniae |
| J14.9 | Pneumonia due to Haemophilus influenzae |
| J15.0 | Pneumonia due to Klebsiella pneumoniae |
| J15.1 | Pneumonia due to Pseudomonas |
| J15.2 | Pneumonia due to staphylococcus |
| J15.4 | Pneumonia due to other streptococci |
| J15.5 | Pneumonia due to Escherichia coli |
| J15.6 | Pneumonia due to other Gram-negative bacteria |
| J15.8 | Other bacterial pneumonia |
| J15.9 | Bacterial pneumonia, unspecified |
| J16.8 | Pneumonia due to other specified infectious organisms |
| J17.1 | Pneumonia in viral diseases classified elsewhere |
| J17.2 | Pneumonia in mycoses |
| J18.0 | Bronchopneumonia, unspecified |
| J18.1 | Lobar pneumonia, unspecified |
| J18.8 | Other pneumonia, organism unspecified |
| J18.9 | Pneumonia, unspecified |
| J20.0 | Acute bronchitis due to Mycoplasma pneumoniae |
| J20.1 | Acute bronchitis due to Haemophilus influenzae |
| J20.2 | Acute bronchitis due to streptococcus |
| J20.5 | Acute bronchitis due to respiratory syncytial virus |
| J20.6 | Acute bronchitis due to rhinovirus |
| J20.9 | Acute bronchitis, unspecified |
| J21.0 | Acute bronchitis due to respiratory syncytial virus |
| J21.8 | Acute bronchiolitis due to other specified organisms |
| J21.9 | Acute bronchitis, unspecified |
| J22.9 | Unspecified acute lower respiratory infection |
| J34.8 | Other specified disorders of nose and nasal sinuses |
| J39.8 | Other specified diseases of upper respiratory tract |
| J40.9 | Bronchitis not specified as acute or chronic |
| J41.0 | Simple chronic bronchitis |
| J44.0 | Chronic obstructive pulmonary disease with acute lower respiratory infection |
| J44.1 | Chronic obstructive pulmonary disease with acute exacerbation, unspecified |
| J44.8 | Other specified chronic obstructive pulmonary disease |
| J45.0A | Asthma bronchiale, allergic asthma, Acute, infectious |
| J45.1A | Asthma bronchiale, nonallergic asthma, Acute, infectious |
| J69.0 | Pneumonitis due to food and vomit |
| J69.8 | Pneumonitis due to other solids and liquids |
| J80.9 | Adult respiratory distress syndrome |
| J80.9B | Adult respiratory distress syndrome, moderate |
| J80.9C | Adult respiratory distress syndrome, severe |
| J80.9X | Adult respiratory distress syndrome, unknown severity |
| J85.0 | Gangrene and necrosis of lung |
| J85.1 | Abscess of lung with pneumonia |
| J86.0 | Pyrothorax with fistula |
| J86.9 | Pyrothorax without fistula |
| J91.9 | Pleural effusions in conditions classified elsewhere |
| J95.8A | Ventilator-associated pneumonia |
| J96.0 | Acute respiratory failure |
| J96.00 | Acute respiratory failure, hypoxia without hypercapnia |
| J96.01 | Acute respiratory failure, hypoxia with hypercapnia |
| J96.09 | Acute respiratory failure, type unspecified |
| J96.9 | Respiratory failure, unspecified |
| J96.90 | Respiratory failure, unspecified, hypoxia without hypercapnia |
| J96.91 | Respiratory failure, unspecified, hypoxia with hypercapnia |
| J96.99 | Respiratory failure, unspecified, type unspecified |
| R05.9 | Cough |
| R06.0 | Dyspnoea |
| R09.2 | Respiratory arrest |
| R50.2 | Pain, not elsewhere classified |
| R50.8 | Other specified fever |
| R50.9 | Fever, unspecified |
| R56.0 | Febrile convulsions |
| R57.2 | Septic shock |
| R57.9 | Shock, unspecified |
| R65.1 | Systemic Inflammatory Response Syndrome of infectious origin with organ failure |
| R91.9 | Abnormal findings on diagnostic imaging of lung |
| U07.1 | COVID-19, virus identified |
| U07.2 | COVID-19, virus not identified |
| U08.9 | Personal history of COVID-19, unspecified |
| U09.9 | Post COVID-19 condition |
| U10.9 | Multisystem inflammatory syndrome associated with COVID-19 |

**Note:** Only main diagnosis codes were considered

**Abbreviations:** COVID-19=Coronavirus disease 2019, ICD-10=International Statistical Classification of Diseases and Related Health Problems 10th Revision

**Table S3. Descriptions of study outcomes and other collected variables**

| **Variable** | **Data sources** | **Missing data** | **Definition** | **Time period** | **Possible values** |
| --- | --- | --- | --- | --- | --- |
| **Study outcomes** | | | | | |
| 30-day all-cause mortality | VAL | No | Date of death (of any cause) any time from day of ED visit and 30 days onwards | 0 to 30 days from day of ED visit | Yes, No |
| 90-day all-cause mortality | VAL | No | Date of death (of any cause) any time from day of ED visit and 90 days onwards | 0 to 90 days from day of ED visit | Yes, No |
| Hospital admission | VAL | No | Admission to the hospital any time from day of ED visit and 14 days onwards. Only hospital admissions with an ICD-10 main diagnosis code indicative of a respiratory virus infection were considered (see Table S2). | 0 to 14 days from day of ED visit | Yes, No |
| ICU admission | SIR | No | Admission to the ICU during a hospital admission as defined above. | Entire hospitalization | Yes, No |
| **Other collected variables** | | | | | |
| Biological sex | VAL | No | Sex of individual | Birth | Male, Female |
| Age | VAL | No | Age at ED visit | Date of ED visit | 18 to 106 years |
| Age category | VAL | No | Age category at ED visit | Date of ED visit | 18 to 44 years, 45 to 54 years, 55 to 64 years, 65 to 74 years, 75 years or older |
| Region of birth | Statistics Sweden | Yes, for 3 individuls | According to the United Nations geoscheme | Birth | Africa, The Americas, Asia or Oceania, Europe, Sweden, Missing |
| Education level | Statistics Sweden | Yes, for 605 individuals | The highest level of completed education in year 2019 | 2019 | Primary, Secondary, Tertiary, Missing |
| COVID-19 vaccination status | NVR | No | Number of COVID-19 vaccine doses received any time up until 14 days before the ED visit | 27 December 2020 up until 14 days before the ED visit | Unvaccinated or 1 dose, 2 doses, 3 doses, 4 doses |
| Previous SARS-CoV-2 infection | SmiNet | No | A positive SARS-CoV-2 PCR-test more than 90 days before the ED visit or a positive SARS-CoV-2 serology test any time up to 14 days before the ED visit and before the first COVID-19 vaccination dose (if any) | 90 days or more before the ED visit | Yes, No |
| Cancer | VAL | No | **ICD-10:** All codes from C00.X to C97.X (besides C44.X), Z51.0, Z51.1  **KVÅ:** DT107, DT108, DT112, DT116, DT135, DV070, DV071, DV134 | Fourteen days to one year before ED visit date  Fourteen days to one year before ED visit date | Yes, No |
| Cardiac or cerebrovascular disease | VAL | No | **ICD-10:** I20.X, I21.X, I22.X, I23.X, I24.X, I25.X, I26.X, I27.X, I42.X, I48.X, I50.X, I61.X, I63.X, I64.X | Fourteen days to five years before ED visit date | Yes, No |
| Chronic kidney failure | VAL | No | **ICD-10:** N18.4, N18.5  **ICD-10:** Z49.1, Z49.2 (should be registered at least twelve times during the time period)  **ICD-10:** Z99.2    **KVÅ:** DR016, DR024 (should be registered at least twelve times during the time period) | Fourteen days to five years before ED visit date  Fourteen days to one year before ED visit date  Fourteen days to one year before ED visit date  Fourteen days to one year before ED visit date | Yes, No |
| Chronic liver disease | VAL | No | **ICD-10:** B18.X, K70.X, K71.7, K72.X, K74.6, K75.X | Fourteen days to five years before ED visit date | Yes, No |
| Chronic lung disease | VAL | No | **ICD-10:** D86.0, D86.2, E84.X, J43.X, J44.X, J46.X, J47.X, J70.3, J84.X, J98.2 | Fourteen days to five years before ED visit date | Yes, No |
| Diabetes (type 1 or 2) | VAL | No | **ICD-10:** E10.X, E11.X | Fourteen days to five years before ED visit date | Yes, No |
| Home care services or nursing home | VAL | No | Receiving home care services or living in a nursing home | Receiving home care services any time up until 14 days before the ED visit  Moving to a nursing home any time up until 14 days before the ED visit | Yes, No |
| Hypertension | VAL | No | **ICD-10:** I10.X, I11.X, I12.X, I13.X, I14.X, I15.X (should be registered at least twice during the time period) | Fourteen days to five years before ED visit date | Yes, No |
| Immunocompromised state | VAL | No | **ATC:** H02AB.X (should be registered at least twice during the time period)  **ATC:** L01.X  **ATC:** L04.X  **ICD-10:** B20.X, B21.X, B22.X, B23.X, B24.X, D57.0, D57.1, D80.X, D81.X, D82.X, D83.X, D84.X, Z94.0, Z94.1, Z94.2, Z94.3, Z94.4, Z94.8  **KVÅ:** DR04.1, DR04.2, DR04.4, DR04.6, DR04.7  **KVÅ:** H02AB.X (should be registered at least twice during the time period)  **KVÅ:** L01.X  **KVÅ:** L04.X | Fourteen days to half a year before ED visit date  Fourteen days to one year before ED visit date  Fourteen days to half a year before ED visit date  Fourteen days or more before ED visit date  Fourteen days to three years before ED visit date  Fourteen days to half a year before ED visit date  Fourteen days to one year before ED visit date  Fourteen days to half a year before ED visit date | Yes, No |
| Mental health disorder | VAL | No | **ICD-10:** F20.X-F29.X, F31.X | Fourteen days to five years before ED visit date | Yes, No |
| Neurological conditions including dementia | VAL | No | **ICD-10:** F00.X, F01.X, F02.X, F03.X, G10.X, G12.2, G20.X, G30.X, G35.X, G70.X, G71.X, G80.X | Fourteen days to five years before ED visit date | Yes, No |
| Obesity | VAL | No | **ICD-10:** E66.X | Fourteen days to five years before ED visit date | Yes, No |

**Abbreviations:** ATC=Anatomical Therapeutic Chemical, COVID-19=Coronavirus disease 2019, ED=Emergency department, ICD-10=International Statistical Classification of Diseases and Related Health Problems 10th Revision, KVÅ=Klassifikation av vårdåtgärder (Swedish for classification of healthcare procedures), PCR=Polymerase chain reaction

**Figure S1. Distribution of circulating Omicron sublineages in Stockholm County over the Omicron study period**

**
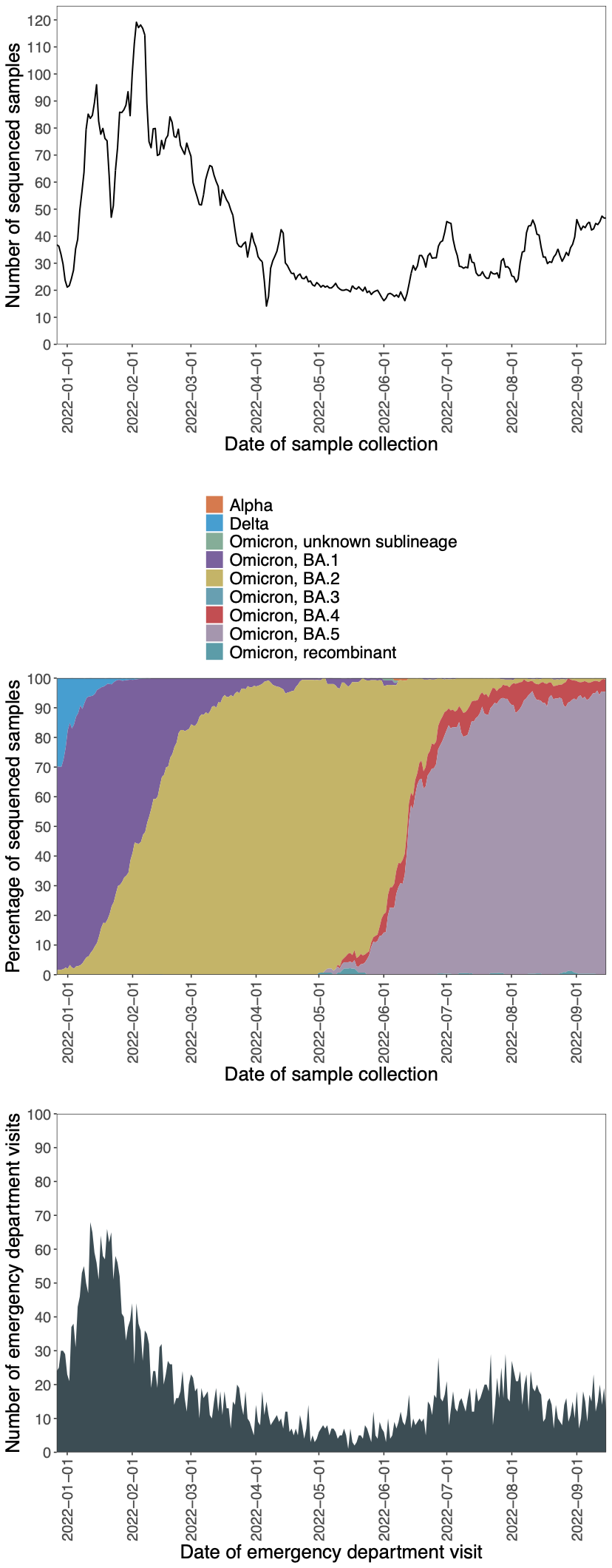
**

**Note:** The top panel shows the 7-day rolling average of the number of sequenced samples in Stockholm. The middle panel shows the Omicron sublineage distribution. The bottom panel shows the main Omicron cohort. The rolling averages were right-aligned. The sequence data was based on metadata from GISAID.

**Table S4. Characteristics of the SARS-CoV-2 omicron study cohort by COVID-19 vaccination status**

| **Variable** | **Unvaccinated**  **(n=1,068)** | **One dose (n=140)** | **Two doses (n=1,128)** | **Three doses (n=1,740)** | **Four doses (n=757)** |
| --- | --- | --- | --- | --- | --- |
| Male sex | 501 (46.9) | 60 (42.9) | 546 (48.4) | 932 (53.6) | 420 (55.5) |
| Age, years | 56.0 [35.0, 74.0] | 49.0 [31.0, 71.2] | 56.0 [39.0, 74.0] | 75.5 [62.0, 83.0] | 81.0 [74.0, 87.0] |
| 18-44 | 391 (36.6) | 61 (43.6) | 365 (32.4) | 137 (7.9) | 6 (0.8) |
| 45-54 | 122 (11.4) | 18 (12.9) | 169 (5.0) | 120 (6.9) | 11 (1.5) |
| 55-64 | 155 (14.5) | 7 (5.0) | 175 (15.5) | 264 (15.2) | 35 (4.6) |
| 65-74 | 145 (13.6) | 24 (17.1) | 153 (13.6) | 306 (17.6) | 139 (18.4) |
| 75 or older | 255 (23.9) | 30 (21.4) | 266 (23.6) | 913 (52.5) | 566 (74.8) |
| Region of birth |  |  |  |  |  |
| Africa | 76 (7.1) | 9 (6.4) | 60 (5.3) | 21 (1.2) | 10 (1.3) |
| The Americas | 46 (4.3) | 6 (4.3) | 38 (3.4) | 39 (2.2) | 10 (1.3) |
| Asia or Oceania | 252 (23.6) | 36 (25.7) | 229 (20.3) | 161 (9.3) | 27 (3.6) |
| Europe | 226 (21.2) | 16 (11.4) | 125 (11.1) | 213 (12.2) | 71 (9.4) |
| Sweden | 468 (43.8) | 73 (52.1) | 676 (59.9) | 1,306 (75.1) | 639 (84.4) |
| Education level |  |  |  |  |  |
| Primary | 314 (29.4) | 48 (34.3) | 282 (25.0) | 404 (23.3) | 200 (26.4) |
| Secondary | 394 (36.9) | 48 (34.3) | 431 (38.2) | 698 (40.1) | 305 (40.3) |
| Tertiary | 265 (24.8) | 32 (22.9) | 334 (29.6) | 579 (33.3) | 234 (30.9) |
| Missing | 95 (8.9) | 12 (8.6) | 81 (7.2) | 59 (3.4) | 18 (2.4) |
| Cancer | 49 (4.6) | 9 (6.4) | 104 (9.2) | 249 (14.3) | 142 (18.8) |
| Cardiac or cerebrovascular disease | 233 (21.8) | 29 (20.7) | 295 (26.2) | 755 (43.4) | 410 (54.2) |
| Chronic kidney failure | 84 (7.9) | 12 (8.6) | 89 (7.9) | 314 (18.0) | 180 (23.8) |
| Chronic liver disease | 21 (2.0) | 2 (1.4) | 47 (4.2) | 48 (2.8) | 15 (2.0) |
| Chronic lung disease | 94 (8.8) | 17 (12.1) | 152 (13.5) | 286 (16.4) | 148 (19.6) |
| Diabetes | 170 (15.9) | 20 (14.3) | 212 (18.8) | 432 (24.8) | 175 (23.1) |
| Home care services or nursing home | 159 (14.9) | 25 (17.9) | 212 (18.8) | 603 (34.7) | 328 (43.3) |
| Hypertension | 333 (31.2) | 45 (32.1) | 410 (36.3) | 1,014 (58.3) | 534 (70.5) |
| Immunosuppression | 102 (9.6) | 13 (9.3) | 159 (14.1) | 441 (25.3) | 187 (24.7) |
| Neurologic conditions, including dementia | 68 (6.4) | 7 (5.0) | 86 (7.6) | 252 (14.5) | 135 (17.8) |
| Obesity | 105 (9.8) | 17 (12.1) | 136 (12.1) | 155 (8.9) | 48 (6.3) |
| Previous SARS-CoV-2 infection | 96 (9.0) | 15 (10.7) | 113 (10.0) | 119 (6.8) | 46 (6.1) |

**Abbreviations:** COVID-19=Coronavirus disease 2019, SARS-CoV-2=Severe acute respiratory syndrome coronavirus 2

**Table S5. Baseline characteristics of individuals with and without an ED diagnosis code indicative of respiratory infection in the Omicron cohort**

| **Variable** | **Overall (n=6,536)** | **No diagnosis**  **(n=1,852)** | **Diagnosis**  **(n=4,684)** | ***P* value** |
| --- | --- | --- | --- | --- |
| Male sex | 3,368 (51.5) | 985 (53.2) | 2,383 (50.9) | 0.098 |
| Age, years | 70.0 [50.0, 81.0] | 70.0 [49.0, 81.0] | 70.0 [50.0, 81.0] | 0.745 |
| 18-44 | 1,321 (20.2) | 383 (20.7) | 938 (20.0) | 0.276 |
| 45-54 | 614 (9.4) | 186 (10.0) | 428 (9.1) |  |
| 55-64 | 831 (12.7) | 211 (11.4) | 620 (13.2) |  |
| 65-74 | 1,041 (15.9) | 292 (15.8) | 749 (16.0) |  |
| 75 or older | 2,729 (41.8) | 780 (42.1) | 1,949 (41.6) |  |
| Region of birth |  |  |  | 0.005 |
| Africa | 224 (3.4) | 53 (2.9) | 171 (3.7) |  |
| The Americas | 185 (2.8) | 49 (2.6) | 136 (2.9) |  |
| Asia or Oceania | 907 (13.9) | 222 (12.0) | 685 (14.6) |  |
| Europe | 892 (13.6) | 262 (14.1) | 630 (13.5) |  |
| Sweden | 4,326 (66.2) | 1,264 (68.3) | 3,062 (65.4) |  |
| Missing | 2 (0.0) | 2 (0.1) | 0 (0.0) |  |
| Education level |  |  |  | 0.386 |
| Primary | 1,650 (25.2) | 448 (24.2) | 1,202 (25.7) |  |
| Secondary | 2,579 (39.5) | 759 (41.0) | 1,820 (38.9) |  |
| Tertiary | 1,958 (30.0) | 551 (29.8) | 1,407 (30.0) |  |
| Missing | 349 (5.3) | 94 (5.1) | 255 (5.4) |  |
| Cancer | 732 (11.2) | 192 (10.4) | 540 (11.5) | 0.194 |
| Cardiac or cerebrovascular disease | 2,302 (35.2) | 657 (35.5) | 1,645 (35.1) | 0.808 |
| Chronic kidney failure | 903 (13.8) | 253 (13.7) | 650 (13.9) | 0.851 |
| Chronic liver disease | 197 (3.0) | 72 (3.9) | 125 (2.7) | 0.012 |
| Chronic lung disease | 856 (13.1) | 194 (10.5) | 662 (14.1) | <0.001 |
| Diabetes | 1,368 (20.9) | 403 (21.8) | 965 (20.6) | 0.316 |
| Home care services or nursing home | 1,753 (26.8) | 479 (25.9) | 1,274 (27.2) | 0.286 |
| Hypertension | 3,159 (48.3) | 915 (49.4) | 2,244 (47.9) | 0.287 |
| Immunocompromised state | 1,101 (16.8) | 230 (12.4) | 871 (18.6) | <0.001 |
| Neurologic conditions, including dementia | 749 (11.5) | 223 (12.0) | 526 (11.2) | 0.376 |
| Obesity | 587 (9.0) | 147 (7.9) | 440 (9.4) | 0.071 |
| COVID-19 vaccine doses |  |  |  | 0.001 |
| Unvaccinated | 1,358 (20.8) | 324 (17.5) | 1,034 (22.1) |  |
| 1 dose | 188 (2.9) | 52 (2.8) | 136 (2.9) |  |
| 2 doses | 1,551 (23.7) | 450 (24.3) | 1,101 (23.5) |  |
| 3 doses | 2,416 (37.0) | 730 (39.4) | 1,686 (36.0) |  |
| 4 doses | 1,023 (15.7) | 296 (16.0) | 727 (15.5) |  |
| Previous SARS-CoV-2 infection | 554 (8.5) | 183 (9.9) | 371 (7.9) | 0.012 |

**Note:** Age was compared with Kruskal Wallis, whereas the other variables were compared with Chi-square tests.

**Abbreviations:** COVID-19=Coronavirus disease 2019, ED=Emergency department, ICU=Intensive care unit, SARS-CoV-2=Severe acute respiratory syndrome coronavirus 2

**Table S6. Characteristics of individuals with and without an ED diagnosis code indicative of respiratory infection in the influenza 2021/2022 cohort**

| **Variable** | **Overall (n=1,289)** | **No diagnosis**  **(n=197)** | **Diagnosis**  **(n=1,092)** | ***P* value** |
| --- | --- | --- | --- | --- |
| Male sex | 549 (42.6) | 80 (40.6) | 469 (42.9) | 0.594 |
| Age, years | 57.0 [32.0, 77.0] | 59.0 [34.0, 78.0] | 57.0 [32.0, 77.0] | 0.578 |
| 18-44 | 524 (40.7) | 76 (38.6) | 448 (41.0) | 0.874 |
| 45-54 | 96 (7.4) | 14 (7.1) | 82 (7.5) |  |
| 55-64 | 126 (9.8) | 21 (10.7) | 105 (9.6) |  |
| 65-74 | 171 (13.3) | 24 (12.2) | 147 (13.5) |  |
| 75 or older | 372 (28.9) | 62 (31.5) | 310 (28.4) |  |
| Region of birth |  |  |  | 0.693 |
| Africa | 77 (6.0) | 11 (5.6) | 66 (6.0) |  |
| The Americas | 48 (3.7) | 7 (3.6) | 41 (3.8) |  |
| Asia or Oceania | 286 (22.2) | 38 (19.3) | 248 (22.7) |  |
| Europe | 141 (10.9) | 19 (9.6) | 122 (11.2) |  |
| Sweden | 737 (57.2) | 122 (61.9) | 615 (56.3) |  |
| Education level |  |  |  | 0.617 |
| Primary | 385 (29.9) | 60 (30.5) | 325 (29.8) |  |
| Secondary | 482 (37.4) | 66 (33.5) | 416 (38.1) |  |
| Tertiary | 353 (27.4) | 60 (30.5) | 293 (26.8) |  |
| Missing | 69 (5.4) | 11 (5.6) | 58 (5.3) |  |
| Cancer | 97 (7.5) | 14 (7.1) | 83 (7.6) | 0.924 |
| Cardiac or cerebrovascular disease | 333 (25.8) | 47 (23.9) | 286 (26.2) | 0.548 |
| Chronic kidney failure | 120 (9.3) | 19 (9.6) | 101 (9.2) | 0.966 |
| Chronic liver disease | 30 (2.3) | 4 (2.0) | 26 (2.4) | 0.965 |
| Chronic lung disease | 163 (12.6) | 15 (7.6) | 148 (13.6) | 0.028 |
| Diabetes | 201 (15.6) | 34 (17.3) | 167 (15.3) | 0.553 |
| Home care services or nursing home | 226 (17.5) | 38 (19.3) | 188 (17.2) | 0.547 |
| Hypertension | 467 (36.2) | 75 (38.1) | 392 (35.9) | 0.614 |
| Immunocompromised state | 143 (11.1) | 18 (9.1) | 125 (11.4) | 0.408 |
| Neurologic conditions, including dementia | 83 (6.4) | 14 (7.1) | 69 (6.3) | 0.797 |
| Obesity | 130 (10.1) | 12 (6.1) | 118 (10.8) | 0.058 |
| COVID-19 vaccine doses |  |  |  | 0.476 |
| Unvaccinated | 243 (18.9) | 33 (16.8) | 210 (19.2) |  |
| 1 dose | 58 (4.5) | 8 (4.1) | 50 (4.6) |  |
| 2 doses | 603 (46.8) | 97 (49.2) | 506 (46.3) |  |
| 3 doses | 351 (27.2) | 57 (28.9) | 294 (26.9) |  |
| 4 doses | 34 (2.6) | 2 (1.0) | 32 (2.9) |  |
| Previous SARS-CoV-2 infection | 275 (21.3) | 35 (17.8) | 240 (22.0) | 0.217 |

**Note:** Age was compared with Kruskal Wallis, whereas the other variables were compared with Chi-square tests.

**Abbreviations:** COVID-19=Coronavirus disease 2019, ED=Emergency department, ICU=Intensive care unit, SARS-CoV-2=Severe acute respiratory syndrome coronavirus 2

**Table S7. Characteristics of individuals with and without an ED diagnosis code indicative of respiratory infection in the RSV 2021/2022 cohort**

| **Variable** | **Overall (n=550)** | **No diagnosis**  **(n=103)** | **Diagnosis**  **(n=447)** | ***P* value** |
| --- | --- | --- | --- | --- |
| Male sex | 260 (47.3) | 58 (56.3) | 202 (45.2) | 0.054 |
| Age, years | 71.0 [53.2, 82.0] | 68.0 [39.5, 81.5] | 72.0 [57.0, 82.0] | 0.059 |
| 18-44 | 103 (18.7) | 32 (31.1) | 71 (15.9) | 0.001 |
| 45-54 | 42 (7.6) | 9 (8.7) | 33 (7.4) |  |
| 55-64 | 63 (11.5) | 10 (9.7) | 53 (11.9) |  |
| 65-74 | 96 (17.5) | 8 (7.8) | 88 (19.7) |  |
| 75 or older | 246 (44.7) | 44 (42.7) | 202 (45.2) |  |
| Region of birth |  |  |  | 0.238 |
| Africa | 20 (3.6) | 5 (4.9) | 15 (3.4) |  |
| The Americas | 8 (1.5) | 0 (0.0) | 8 (1.8) |  |
| Asia or Oceania | 80 (14.5) | 14 (13.6) | 66 (14.8) |  |
| Europe | 88 (16.0) | 11 (10.7) | 77 (17.2) |  |
| Sweden | 354 (64.4) | 73 (70.9) | 281 (62.9) |  |
| Education level |  |  |  | 0.872 |
| Primary | 143 (26.0) | 26 (25.2) | 117 (26.2) |  |
| Secondary | 197 (35.8) | 38 (36.9) | 159 (35.6) |  |
| Tertiary | 180 (32.7) | 35 (34.0) | 145 (32.4) |  |
| Missing | 30 (5.5) | 4 (3.9) | 26 (5.8) |  |
| Cancer | 68 (12.4) | 9 (8.7) | 59 (13.2) | 0.283 |
| Cardiac or cerebrovascular disease | 217 (39.5) | 45 (43.7) | 172 (38.5) | 0.388 |
| Chronic kidney failure | 86 (15.6) | 14 (13.6) | 72 (16.1) | 0.629 |
| Chronic liver disease | 18 (3.3) | 3 (2.9) | 15 (3.4) | 1.000 |
| Chronic lung disease | 122 (22.2) | 8 (7.8) | 114 (25.5) | <0.001 |
| Diabetes | 126 (22.9) | 17 (16.5) | 109 (24.4) | 0.113 |
| Home care services or nursing home | 143 (26.0) | 20 (19.4) | 123 (27.5) | 0.118 |
| Hypertension | 290 (52.7) | 53 (51.5) | 237 (53.0) | 0.859 |
| Immunocompromised state | 101 (18.4) | 10 (9.7) | 91 (20.4) | 0.018 |
| Neurologic conditions, including dementia | 48 (8.7) | 9 (8.7) | 39 (8.7) | 1.000 |
| Obesity | 67 (12.2) | 11 (10.7) | 56 (12.5) | 0.726 |
| COVID-19 vaccine doses |  |  |  | 0.172 |
| Unvaccinated | 60 (10.9) | 15 (14.6) | 45 (10.1) |  |
| 1 dose | 28 (5.1) | 9 (8.7) | 19 (4.3) |  |
| 2 doses | 337 (61.3) | 60 (58.3) | 277 (62.0) |  |
| 3 doses | 123 (22.4) | 19 (18.4) | 104 (23.3) |  |
| 4 doses | 2 (0.4) | 0 (0.0) | 2 (0.4) |  |
| Previous SARS-CoV-2 infection | 92 (16.7) | 17 (16.5) | 75 (16.8) | 1.000 |

**Note:** Age was compared with Kruskal Wallis, whereas the other variables were compared with Chi-square tests.

**Abbreviations:** COVID-19=Coronavirus disease 2019, ED=Emergency department, ICU=Intensive care unit, RSV=Respiratory syncytial virus, SARS-CoV-2=Severe acute respiratory syndrome coronavirus 2

**Table S8. Characteristics of individuals with and without an ED diagnosis code indicative of respiratory infection in the influenza 2015-2019 cohort**

| **Variable** | **Overall (n=6,638)** | **No diagnosis**  **(n=1,017)** | **Diagnosis**  **(n=5,621)** | ***P* value** |
| --- | --- | --- | --- | --- |
| Male sex | 3,054 (46.0) | 461 (45.3) | 2,593 (46.1) | 0.662 |
| Age, years | 68.0 [47.0, 80.0] | 70.0 [50.0, 81.0] | 68.0 [47.0, 80.0] | 0.233 |
| 18-44 | 1,498 (22.6) | 222 (21.8) | 1,276 (22.7) | 0.210 |
| 45-54 | 663 (10.0) | 87 (8.6) | 576 (10.2) |  |
| 55-64 | 795 (12.0) | 112 (11.0) | 683 (12.2) |  |
| 65-74 | 1,150 (17.3) | 188 (18.5) | 962 (17.1) |  |
| 75 or older | 2,532 (38.1) | 408 (40.1) | 2,124 (37.8) |  |
| Region of birth |  |  |  | 0.075 |
| Africa | 275 (4.1) | 40 (3.9) | 235 (4.2) |  |
| The Americas | 174 (2.6) | 25 (2.5) | 149 (2.7) |  |
| Asia or Oceania | 974 (14.7) | 121 (11.9) | 853 (15.2) |  |
| Europe | 919 (13.8) | 135 (13.3) | 784 (13.9) |  |
| Sweden | 4,293 (64.7) | 696 (68.4) | 3,597 (64.0) |  |
| Missing | 3 (0.0) | 0 (0.0) | 3 (0.1) |  |
| Education level |  |  |  | 0.911 |
| Primary | 1,738 (26.2) | 274 (26.9) | 1,464 (26.0) |  |
| Secondary | 2,673 (40.3) | 410 (40.3) | 2,263 (40.3) |  |
| Tertiary | 1,986 (29.9) | 298 (29.3) | 1,688 (30.0) |  |
| Missing | 241 (3.6) | 35 (3.4) | 206 (3.7) |  |
| Cancer | 605 (9.1) | 83 (8.2) | 522 (9.3) | 0.276 |
| Cardiac or cerebrovascular disease | 2,109 (31.8) | 327 (32.2) | 1,782 (31.7) | 0.804 |
| Chronic kidney failure | 525 (7.9) | 84 (8.3) | 441 (7.8) | 0.699 |
| Chronic liver disease | 164 (2.5) | 30 (2.9) | 134 (2.4) | 0.337 |
| Chronic lung disease | 996 (15.0) | 119 (11.7) | 877 (15.6) | 0.002 |
| Diabetes | 1,154 (17.4) | 194 (19.1) | 960 (17.1) | 0.133 |
| Home care services or nursing home | 1,280 (19.3) | 200 (19.7) | 1,080 (19.2) | 0.769 |
| Hypertension | 2,727 (41.1) | 418 (41.1) | 2,309 (41.1) | 1.000 |
| Immunocompromised state | 1,009 (15.2) | 146 (14.4) | 863 (15.4) | 0.443 |
| Neurologic conditions, including dementia | 631 (9.5) | 103 (10.1) | 528 (9.4) | 0.499 |
| Obesity | 437 (6.6) | 57 (5.6) | 380 (6.8) | 0.194 |

**Note:** Age was compared with Kruskal Wallis, whereas the other variables were compared with Chi-square tests.

**Abbreviations:** ED=Emergency department, ICU=Intensive care unit

**Table S9. Characteristics of individuals with and without an ED diagnosis code indicative of respiratory infection in the RSV 2015-2019 cohort**

| **Variable** | **Overall (n=1,157)** | **No diagnosis**  **(n=178)** | **Diagnosis**  **(n=979)** | ***P* value** |
| --- | --- | --- | --- | --- |
| Male sex | 480 (41.5) | 67 (37.6) | 413 (42.2) | 0.294 |
| Age, years | 75.0 [63.0, 84.0] | 69.0 [56.2, 81.0] | 76.0 [63.0, 85.0] | <0.001 |
| 18-44 | 112 (9.7) | 24 (13.5) | 88 (9.0) | <0.001 |
| 45-54 | 63 (5.4) | 15 (8.4) | 48 (4.9) |  |
| 55-64 | 155 (13.4) | 27 (15.2) | 128 (13.1) |  |
| 65-74 | 235 (20.3) | 48 (27.0) | 187 (19.1) |  |
| 75 or older | 592 (51.2) | 64 (36.0) | 528 (53.9) |  |
| Region of birth |  |  |  | 0.695 |
| Africa | 34 (2.9) | 5 (2.8) | 29 (3.0) |  |
| The Americas | 25 (2.2) | 5 (2.8) | 20 (2.0) |  |
| Asia or Oceania | 102 (8.8) | 16 (9.0) | 86 (8.8) |  |
| Europe | 160 (13.8) | 30 (16.9) | 130 (13.3) |  |
| Sweden | 836 (72.3) | 122 (68.5) | 714 (72.9) |  |
| Education level |  |  |  | 0.715 |
| Primary | 345 (29.8) | 56 (31.5) | 289 (29.5) |  |
| Secondary | 454 (39.2) | 72 (40.4) | 382 (39.0) |  |
| Tertiary | 310 (26.8) | 45 (25.3) | 265 (27.1) |  |
| Missing | 48 (4.1) | 5 (2.8) | 43 (4.4) |  |
| Cancer | 174 (15.0) | 27 (15.2) | 147 (15.0) | 1.000 |
| Cardiac or cerebrovascular disease | 524 (45.3) | 65 (36.5) | 459 (46.9) | 0.013 |
| Chronic kidney failure | 131 (11.3) | 16 (9.0) | 115 (11.7) | 0.347 |
| Chronic liver disease | 31 (2.7) | 5 (2.8) | 26 (2.7) | 1.000 |
| Chronic lung disease | 298 (25.8) | 30 (16.9) | 268 (27.4) | 0.004 |
| Diabetes | 253 (21.9) | 41 (23.0) | 212 (21.7) | 0.756 |
| Home care services or nursing home | 343 (29.6) | 45 (25.3) | 298 (30.4) | 0.195 |
| Hypertension | 597 (51.6) | 74 (41.6) | 523 (53.4) | 0.005 |
| Immunocompromised state | 274 (23.7) | 47 (26.4) | 227 (23.2) | 0.405 |
| Neurologic conditions, including dementia | 132 (11.4) | 16 (9.0) | 116 (11.8) | 0.329 |
| Obesity | 81 (7.0) | 17 (9.6) | 64 (6.5) | 0.197 |

**Note:** Age was compared with Kruskal Wallis, whereas the other variables were compared with Chi-square tests.

**Abbreviations:** ED=Emergency department, ICU=Intensive care unit, RSV=Respiratory syncytial virus

**Figure S2. Cumulative incidence plot for 90-day all-cause mortality in the main analysis and the cohorts including all visits**

**
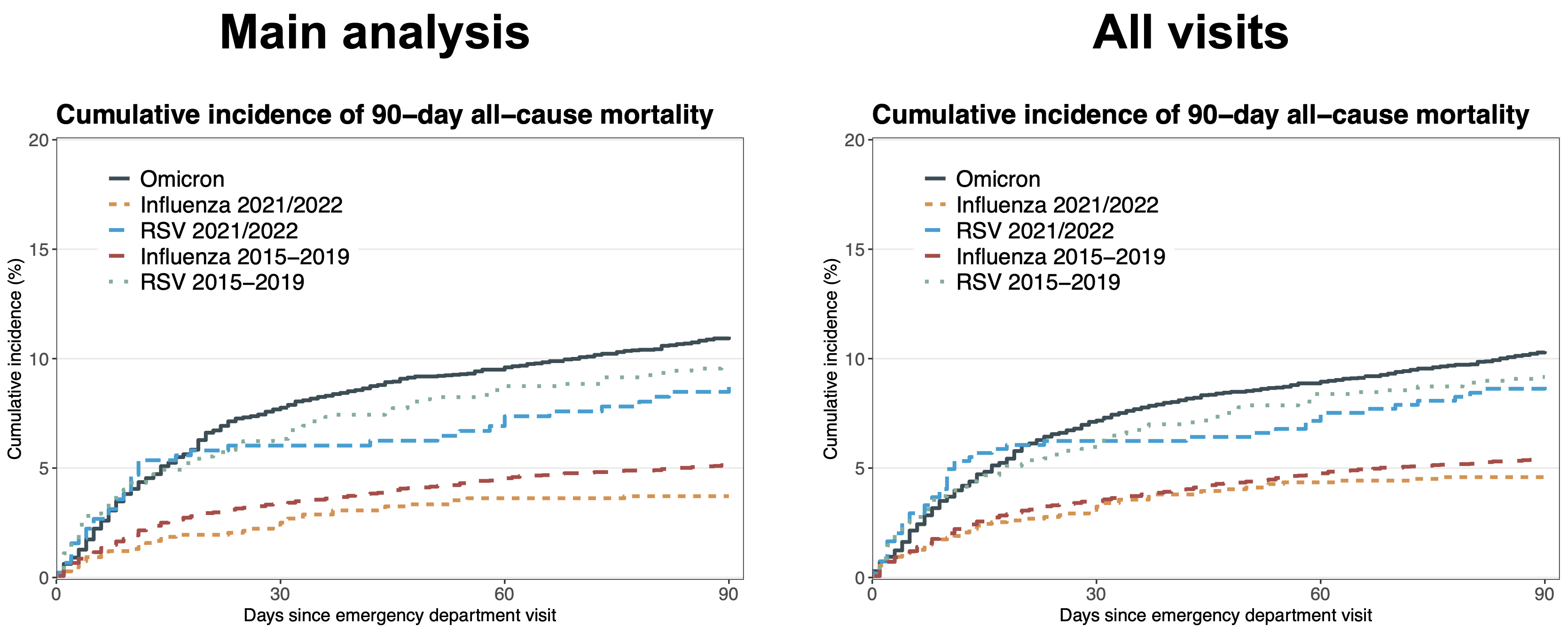
**

**Note:** This analysis was restricted to patients attending the emergency department up until 17 July 2022 to enable for 90 days of follow-up.

**Abbreviations:** RSV=Respiratory syncytial virus

**Table S10. Unadjusted regression model ratios in the main analysis compared with a sensitivity analysis including all visits**

|  | **Omicron versus influenza**  **Unadjusted ratio (95% CI)** | |  | **Omicron versus RSV**  **Unadjusted ratio (95% CI)** | |
| --- | --- | --- | --- | --- | --- |
|  | **Main analysis** | **All visits** |  | **Main analysis** | **All visits** |
| **30-day all-cause mortality ^a^** |  |  |  |  |  |
| Main cohorts | 3.27 (2.26-4.94) | 2.34 (1.72-3.28) |  | 1.35 (0.92-2.07) | 1.20 (0.85-1.75) |
| Unvaccinated Omicron ^b^ | 3.52 (2.31-5.52) | 2.59 (1.81-3.77) |  | 1.45 (0.94-2.30) | 1.32 (0.90-2.00) |
| Vaccinated Omicron ^c^ | 3.24 (2.22-4.91) | 2.30 (1.68-3.23) |  | 1.34 (0.91-2.05) | 1.18 (0.83-1.72) |
| Pre-pandemic influenza and RSV | 2.46 (2.06-2.94) | 2.17 (1.85-2.55) |  | 1.27 (0.97-1.68) | 1.24 (0.97-1.63) |
| **90-day all-cause mortality ^a,d^** |  |  |  |  |  |
| Main cohorts | 3.15 (2.31-4.43) | 2.39 (1.83-3.17) |  | 1.30 (0.93-1.85) | 1.20 (0.89-1.65) |
| Unvaccinated Omicron ^b^ | 2.87 (1.98-4.25) | 2.18 (1.58-3.05) |  | 1.18 (0.81-1.75) | 1.14 (0.82-1.63) |
| Vaccinated Omicron ^c^ | 3.33 (2.40-4.72) | 2.48 (1.88-3.32) |  | 1.35 (0.96-1.93) | 1.23 (0.91-1.71) |
| Pre-pandemic influenza and RSV | 2.28 (1.96-2.67) | 2.02 (1.76-2.32) |  | 1.16 (0.92-1.47) | 1.14 (0.92-1.43) |
| **Hospital admission ^e^** |  |  |  |  |  |
| Main cohorts | 1.37 (1.24-1.52) | 1.18 (1.08-1.30) |  | 0.68 (0.60-0.77) | 0.66 (0.59-0.74) |
| Unvaccinated Omicron ^b^ | 1.21 (1.07-1.38) | 1.11 (0.98-1.25) |  | 0.60 (0.52-0.70) | 0.62 (0.54-0.71) |
| Vaccinated Omicron ^c^ | 1.44 (1.30-1.60) | 1.22 (1.10-1.34) |  | 0.71 (0.63-0.81) | 0.68 (0.60-0.76) |
| Pre-pandemic influenza and RSV | 0.93 (0.88-0.98) | 0.76 (0.72-0.79) |  | 0.64 (0.59-0.70) | 0.56 (0.52-0.61) |
| **ICU admission ^e^** |  |  |  |  |  |
| Main cohorts | 2.93 (1.59-5.42) | 2.67 (1.49-4.80) |  | 1.32 (0.69-2.50) | 1.13 (0.63-2.03) |
| Unvaccinated Omicron ^b^ | 5.16 (2.70-9.87) | 4.50 (2.41-8.39) |  | 2.31 (1.18-4.54) | 1.90 (1.02-3.54) |
| Vaccinated Omicron ^c^ | 2.31 (1.23-4.34) | 2.21 (1.22-4.03) |  | 1.04 (0.54-2.00) | 0.93 (0.51-1.70) |
| Pre-pandemic influenza and RSV | 0.89 (0.71-1.10) | 0.73 (0.59-0.89) |  | 0.57 (0.41-0.78) | 0.50 (0.37-0.68) |

**Abbreviations:** CI=Confidence interval, ICU=Intensive care unit, RSV=Respiratory syncytial virus

a. Analysed with logistic regression models

b. Defined as having received zero COVID-19 vaccine doses fourteen days or more before the emergency department visit

c. Defined as having received 2 or more COVID-19 vaccine doses fourteen days or more before the emergency department visit

d. Restricted to individuals visiting the emergency department up until 17 July 2022 to allow for 90-days of follow-up

e. Analysed with Cox proportional hazard regression models

**Table S11. Adjusted regression model ratios in the main analysis compared with a sensitivity analysis including all visits**

|  | **Omicron versus influenza**  **Adjusted ratio (95% CI)** | |  | **Omicron versus RSV**  **Adjusted ratio (95% CI)** | |
| --- | --- | --- | --- | --- | --- |
|  | **Main analysis** | **All visits** |  | **Main analysis** | **All visits** |
| **30-day all-cause mortality ^b^** |  |  |  |  |  |
| Main cohorts | 2.36 (1.60-3.62) | 1.64 (1.18-2.32) |  | 1.42 (0.94-2.21) | 1.20 (0.83-1.77) |
| Unvaccinated Omicron ^c^ | 5.51 (3.41-9.18) | 3.96 (2.63-6.08) |  | 3.29 (2.01-5.56) | 2.92 (1.88-4.66) |
| Vaccinated Omicron ^d^ | 2.00 (1.35-3.10) | 1.37 (0.98-1.96) |  | 1.20 (0.79-1.88) | 1.01 (0.70-1.51) |
| Pre-pandemic influenza and RSV | 2.17 (1.80-2.62) | 1.90 (1.60-2.25) |  | 1.49 (1.12-2.01) | 1.42 (1.09-1.89) |
| **90-day all-cause mortality ^b,e^** |  |  |  |  |  |
| Main cohorts | 2.31 (1.65-3.30) | 1.69 (1.27-2.29) |  | 1.40 (0.98-2.03) | 1.23 (0.90-1.73) |
| Unvaccinated Omicron ^c^ | 4.94 (3.17-7.88) | 3.64 (2.48-5.42) |  | 2.72 (1.75-4.31) | 2.62 (1.76-3.96) |
| Vaccinated Omicron ^d^ | 2.17 (1.53-3.16) | 1.56 (1.16-2.14) |  | 1.27 (0.88-1.88) | 1.14 (0.82-1.61) |
| Pre-pandemic influenza and RSV | 2.09 (1.77-2.48) | 1.82 (1.57-2.12) |  | 1.43 (1.12-1.85) | 1.38 (1.10-1.76) |
| **Hospital admission ^f^** |  |  |  |  |  |
| Main cohorts | 0.97 (0.88-1.08) | 0.86 (0.78-0.95) |  | 0.73 (0.64-0.82) | 0.68 (0.61-0.77) |
| Unvaccinated Omicron ^c^ | 1.30 (1.14-1.49) | 1.20 (1.06-1.36) |  | 0.92 (0.79-1.08) | 0.90 (0.78-1.05) |
| Vaccinated Omicron ^d^ | 0.90 (0.81-1.00) | 0.79 (0.71-0.87) |  | 0.68 (0.60-0.78) | 0.64 (0.57-0.72) |
| Pre-pandemic influenza and RSV | 0.84 (0.79-0.89) | 0.69 (0.65-0.72) |  | 0.75 (0.69-0.82) | 0.66 (0.61-0.72) |
| **ICU admission ^f^** |  |  |  |  |  |
| Main cohorts | 2.49 (1.34-4.63) | 2.23 (1.24-4.04) |  | 1.40 (0.74-2.67) | 1.19 (0.66-2.14) |
| Unvaccinated Omicron ^c^ | 4.96 (2.56-9.60) | 4.31 (2.29-8.14) |  | 2.82 (1.39-5.73) | 2.18 (1.14-4.19) |
| Vaccinated Omicron ^d^ | 1.88 (0.98-3.61) | 1.73 (0.93-3.20) |  | 1.08 (0.56-2.10) | 0.96 (0.52-1.75) |
| Pre-pandemic influenza and RSV | 0.86 (0.69-1.07) | 0.70 (0.57-0.87) |  | 0.58 (0.41-0.80) | 0.53 (0.39-0.72) |

**Abbreviations:** CI=Confidence interval, ICU=Intensive care unit, RSV=Respiratory syncytial virus

a. Adjusted for age, sex, being born in Sweden, education level, and all studied comorbidities

b. Analysed with logistic regression models

c. Defined as having received zero COVID-19 vaccine doses fourteen days or more before the emergency department visit

d. Defined as having received 2 or more COVID-19 vaccine doses fourteen days or more before the emergency department visit

e. Restricted to individuals visiting the emergency department up until 17 July 2022 to allow for 90-days of follow-up

f. Analysed with Cox proportional hazard regression models

**Supplementary references**

1 Hergens M-P, Bell M, Haglund P, *et al.* Risk factors for COVID-19-related death, hospitalization and intensive care: a population-wide study of all inhabitants in Stockholm. *Eur J Epidemiol* 2022; **37**: 157–65.

2 Hedberg P, Granath F, Bruchfeld J, *et al.* Post COVID‐19 condition diagnosis: A population‐based cohort study of occurrence, associated factors, and healthcare use by severity of acute infection. *J Intern Med* 2023; **293**: 246–58.

3 Statistics Sweden. About Statistics Sweden. Stat. Cent. https://www.scb.se/en/About-us/ (accessed Sept 29, 2022).

4 Rolfhamre P, Janson A, Arneborn M, Ekdahl K. SmiNet-2: Description of an internet-based surveillance system for communicable diseases in Sweden. *Euro Surveill Bull Eur Sur Mal Transm Eur Commun Dis Bull* 2006; **11**: 15—16.

5 Notifiable diseases - The Public Health Agency of Sweden. https://www.folkhalsomyndigheten.se/the-public-health-agency-of-sweden/communicable-disease-control/surveillance-of-communicable-diseases/notifiable-diseases/ (accessed March 9, 2023).

6 Swedish Government. Smittskyddslag (2004:168). https://rkrattsbaser.gov.se/sfst?bet=2004:168 (accessed Sept 29, 2022).

7 Public Health Agency of Sweden. Nationella vaccinationsregistret — Folkhälsomyndigheten. https://www.folkhalsomyndigheten.se/smittskydd-beredskap/vaccinationer/nationella-vaccinationsregistret/ (accessed March 9, 2023).

8 Mårtensson J, Engerström L, Walther S, Grip J, Berggren RK, Larsson E. COVID-19 critical illness in Sweden: characteristics and outcomes at a national population level. *Crit Care Resusc* 2020; **22**: 312–20.

9 Zettersten E, Engerström L, Bell M, *et al.* Long-term outcome after intensive care for COVID-19: differences between men and women—a nationwide cohort study. *Crit Care* 2021; **25**: 86.
